# Type E Botulinum Neurotoxin-Producing Clostridium butyricum Strains Are Aerotolerant during Vegetative Growth

**DOI:** 10.1128/mSystems.00299-18

**Published:** 2019-04-30

**Authors:** Serena Camerini, Lucia Marcocci, Lara Picarazzi, Egidio Iorio, Irene Ruspantini, Paola Pietrangeli, Marco Crescenzi, Giovanna Franciosa

**Affiliations:** a Core Facilities, Istituto Superiore di Sanità, Rome, Italy; b Department of Biochemical Sciences Rossi Fanelli, Sapienza University of Rome, Rome, Italy; c Department of Food Safety, Nutrition and Veterinary Public Health, Istituto Superiore di Sanità, Rome, Italy; NYU School of Medicine

**Keywords:** Clostridium butyricum, botulinum neurotoxin, oxidative stress

## Abstract

Clostridium butyricum, the type species of the genus *Clostridium*, is considered an obligate anaerobe, yet it has been shown to grow in the presence of oxygen. *C. butyricum* strains atypically producing the botulinum neurotoxin type E are the leading cause of type E human botulism in Italy. Here, we show that type E botulinum neurotoxin-producing *C. butyricum* strains growing exponentially were able to keep growing and producing toxin *in vitro* upon exposure to air, although less efficiently than under ideal oxygen-depleted conditions. Bacterial growth in air was maintained when the initial cell density was higher than 10^3^ cells/ml. No spores were detected in the cultures aerated for 5 h. To understand the biological mechanisms allowing the adaptation of vegetative cells of *C. butyricum* type E to oxygen, we compared the proteome and metabolome profiles of the clostridial cultures grown for 5 h under either aerated or anaerobic conditions. The results indicated that bacterial cells responded to oxygen stress by slowing growth and modulating the expression of proteins involved in carbohydrate uptake and metabolism, redox homeostasis, DNA damage response, and bacterial motility. Moreover, the ratio of acetate to butyrate was significantly higher under aeration. This study demonstrates for the first time that a botulinum neurotoxin-producing *Clostridium* can withstand oxygen during vegetative growth.

**IMPORTANCE** Botulinum neurotoxins, the causative agents of the potentially fatal disease of botulism, are produced by certain *Clostridium* strains during vegetative growth, usually in anaerobic environments. Our findings indicate that, contrary to current understanding, the growth of neurotoxigenic *C. butyricum* strains and botulinum neurotoxin type E production can continue upon transfer from anaerobic to aerated conditions and that adaptation of strains to oxygenated environments requires global changes in proteomic and metabolic profiles. We hypothesize that aerotolerance might constitute an unappreciated factor conferring physiological advantages on some botulinum toxin-producing clostridial strains, allowing them to adapt to otherwise restrictive environments.

## Abstract (precis)

Botulinum neurotoxins, the causative agents of the potentially fatal disease of botulism, are produced by certain *Clostridium* strains during vegetative growth, usually in anaerobic environments. Our findings indicate that, contrary to current understanding, the growth of neurotoxigenic *C. butyricum* strains and botulinum neurotoxin type E production can continue upon transfer from anaerobic to aerated conditions and that adaptation of strains to oxygenated environments requires global changes in proteomic and metabolic profiles. We hypothesize that aerotolerance might constitute an unappreciated factor conferring physiological advantages on some botulinum toxin-producing clostridial strains, allowing them to adapt to otherwise restrictive environments.

## INTRODUCTION

Bacteria of the genus *Clostridium* are conventionally defined as obligate anaerobes, i.e., they have an oxygen-independent metabolism and could be killed by exposure to oxygen or form resistant spores that germinate into vegetative cells when the conditions become favorable. During vegetative growth, the toxigenic clostridial species produce toxins. Therefore, although clostridial spores are commonly isolated from environments and food products in normal atmospheric oxygen tensions, spore germination and vegetative growth—and toxin production by toxigenic species—occur only in the absence of oxygen (1).

However, while the germination of clostridial spores is rare in the presence of low oxygen concentrations, evidence suggests that vegetative cells may display considerable ability to tolerate oxygen (2, 3).

Based on current approaches, different levels of oxygen tolerance during the vegetative stage have been reported in strains of Clostridium butyricum (2, 4), which is the type species of the genus, as well as in other nontoxigenic *Clostridium* species, including C. acetobutylicum, C. aminovalericum, C. bifermentans, C. puniceum, C. ljungdahlii, C. tertium, and C. glycolicum (5–12), and—of critical importance to human health—in toxigenic species such as C. difficile and C. perfringens (13–15).

Oxygen tolerance among clostridia has been attributed to the enzymatic ability of strains to consume oxygen from the medium and to defend themselves against the toxic effects of the reactive oxygen species (ROS) (2, 4, 16). Recently, a strategy based on the synthesis of aromatic polyketides (clostrubins) has been proposed for the plant pathogen *C. puniceum* to survive and grow in aerated environments; nonetheless, the antioxidant role of clostrubins has not been determined (9).

Certain clostridial strains produce the botulinum neurotoxin (BoNT): this protein toxin usually causes severe paralysis in humans when it is synthesized by BoNT-producing clostridia in the colonized intestine, especially in infants younger than 1 year (infant botulism); in infected wounds (wound botulism); or in contaminated food products before consumption (foodborne botulism) (17). Oxygen may not be completely absent from these environments. The newborn intestine is known to be aerobic until it is made anaerobic by oxygen-reducing aerobes (18), wounds come in contact with ambient air, and even the contaminated food products at risk of botulism may be subject to air infiltration. Therefore, studying the responses of BoNT-producing clostridia to oxygen exposure, especially in the vegetative growth phase when they produce BoNT, is imperative for better understanding the within-host dynamics and implementing food safety control measures.

Type E BoNT (BoNT/E), i.e., one of the antigenically different BoNT types causing human botulism, is usually synthesized by C. botulinum type E strains but can also be produced by atypical neurotoxigenic *C. butyricum* type E strains (17). In Italy, where neurotoxigenic *C. butyricum* type E strains were first isolated from infants with botulism and then repeatedly recovered from cases of human botulism, these strains appear to be clinically more relevant than C. botulinum type E strains (19). Moreover, neurotoxigenic *C. butyricum* type E strains have been associated with human botulism in Asia, the United Kingdom, and the United States, contributing to the reemergence of this microorganism as a causative agent of botulism (20–23).

Although it has been reported that nonneurotoxigenic *C. butyricum* strains grow in oxygen-containing environments, little is known about the behavior of neurotoxigenic *C. butyricum* type E strains upon oxygen exposure. The present study aimed to investigate the effects of atmospheric oxygen exposure on the vegetative growth of neurotoxigenic *C. butyricum* type E strains and BoNT/E production and characterize the strategic defense mechanisms adopted by these microorganisms upon air exposure.

## RESULTS

### Effects of air exposure on the vegetative growth of neurotoxigenic *C. butyricum* type E strains and BoNT/E production.

The broth cultures of the *C. butyricum* type E strains ISS-21 and ISS-190 in the mid-exponential-growth phase were analyzed in parallel during a 5-h incubation either under anaerobic (AN) conditions or following ambient air exposure (aerated [AE] conditions).

At the end of the experiments, the average concentrations of dissolved oxygen in AE cultures were 5.2 ± 1 ppm, whereas oxygen was not detectable in AN cultures. The average pH was 5.35 ± 0.15 in both AN and AE cultures.

**FIG 1:**
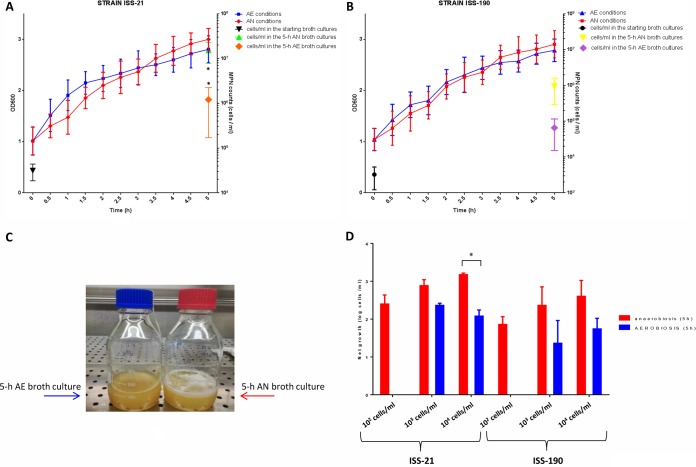
Growth of neurotoxigenic *C. butyricum* type E strains ISS-21 and ISS-190 under anaerobic (AN) or aerated (AE) conditions. (A and B) Growth curves of strains ISS-21 (A) and ISS-190 (B) were generated by measuring the OD_600_ values. The number of viable cells (symbols) was determined in the starting (mid-exponential-phase) broth cultures and after a 5-h incubation under AN or AE conditions. (C) At the end of the experiments, foam was visible in the AN broth cultures but not in the AE broth cultures. (D) Net growth of the *C. butyricum* type E strains ISS-21 and ISS-190 at different initial bacterial densities after a 5-h incubation under AN or AE conditions. Under AE conditions, net growth was observed only when the initial densities were >10^3^ cells/ml. The errors were calculated by determining the standard deviation from the mean for three independent experiments for each strain. *, *P* < 0.05 according to Student’s *t* test.

The two strains exhibited similar OD_600_ growth curves under AN and AE conditions over the 5-h culture period, with an overall ∼2-fold increase in the OD_600_ values and no significant differences between each tested time point (Fig. 1A and B). As the OD_600_ values measure the turbidity of bacterial suspensions regardless of cell viability, the viable cells in the starting (mid-exponential-growth-phase) cultures and final cultures in AN and AE environments were counted. The average cell count of strains ISS-190 and ISS-21 in the starting cultures was ∼10^3^ and 10^4^ cells/ml, respectively (Fig. 1A and B). After 5 h of incubation under AN conditions, the average viable cell count in strains ISS-190 and ISS-21 was 9.4 × 10^5^ cells/ml and 1.5 × 10^7^ cells/ml, respectively; in contrast, the average viable cell count in strains ISS-190 and ISS-21 after a 5-h incubation under AE conditions was 6.5 × 10^4^ and 1.2 × 10^6^ cells/ml, respectively. Therefore, the number of viable cells at the end of the experiments was higher in AN cultures than in AE cultures, and the difference in the average viable cell count under AN and AE conditions was significant only for strain ISS-21 (*P* < 0.05) (Fig. 1A and B). In addition, the average cell count at the end of the experiments was significantly higher in strain ISS-21 than in strain ISS-190 in the AN environment, whereas there was no statistically significant difference between the strains in the AE environment.

Of note is that, at the end of the growth period, gas bubbles were visible in strains cultivated under AN conditions but were not visible in strains grown under AE conditions (Fig. 1C). Importantly, no spores were detected by staining or heat shock in both neurotoxigenic *C. butyricum* type E strains in the AE environment (data not shown).

Since it is known that bacteria respond to changes in environmental conditions in a cell-density-dependent manner (24), we determined the minimum initial bacterial density necessary to promote growth under AE conditions. The results indicated that both *C. butyricum* type E strains could grow under AE conditions when the initial density was at least 10^3^ cells/ml of bacteria growing exponentially (Fig. 1D).

**FIG 2:**
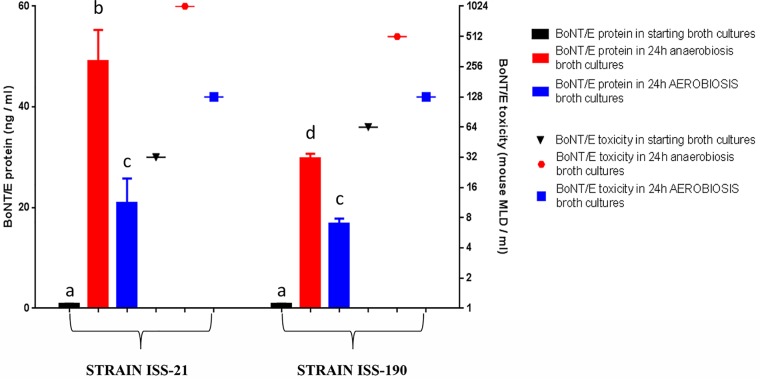
BoNT/E protein and toxicity levels in the starting (mid-exponential-phase) broth cultures of *C. butyricum* type E strains ISS-21 and ISS-190 and in the cultures after 24 h of incubation under AN or AE conditions. BoNT/E protein levels (ng/ml) were measured by ELISA (histogram bars); BoNT/E toxicity levels (mouse lethal dose [MLD]/ml) were assayed by mouse test (symbols). Data are the mean values from two independent experiments for each strain, with samples assayed in duplicate in each ELISA. Columns that do not share the same letter are statistically different according to Student’s *t* test (*P* < 0.05).

With regard to BoNT/E production, BoNT/E protein and toxicity levels were measured in the starting cultures of both strains and in the cultures maintained in the AN environment for 24 h (when the BoNT/E levels should be highest) (25) or incubated in the AE environment for the same time. As expected, BoNT/E protein levels determined by an immuno-ELISA were low in the starting cultures, with no significant differences between the two strains (Fig. 2). The production of BoNT/E protein was increased approximately 58-fold and 36-fold in strains ISS-21 and ISS-190, respectively, after culturing under AN conditions for 24 h and approximately 25-fold and 20-fold in the same strains under AE conditions for 24 h. Therefore, the increase in BoNT/E protein levels in the AE environment was significantly lower than in the AN environment for both strains (*P* < 0.05). Furthermore, while the BoNT/E protein levels were significantly higher in strain ISS-21 than in strain ISS-190 under AN conditions for 24 h (*P* < 0.05), the BoNT/E protein levels were similar between the two strains under AE conditions for 24 h (Fig. 2). The BoNT/E toxicity levels measured using a mouse bioassay were increased 32-fold and 8-fold in strains ISS-21 and ISS-190 cultured under AN conditions for 24 h, respectively, and 4-fold and 2-fold in the same strains under AE conditions for 24 h, respectively (Fig. 2). The increase in BoNT/E protein and toxicity levels was more evident in strain ISS-21 than in strain ISS-190, especially in the AN environment, and this result is consistent with the significantly higher growth rate of the former strain under these culture conditions.

### Effects of air exposure on protein expression.

As *C. butyricum* type E strain ISS-190 appeared less affected by air exposure than strain ISS-21, considering the more similar growth characteristics and BoNT/E production under AE and AN conditions, strain ISS-190 was selected for the comparative proteomics analysis. The *C. butyricum* type E strains ISS-190 and ISS-21 are clonally related, with most of the genetic diversity between the strains consisting of an ∼168-kb genetic region that is present in the ISS-190 genome but missing from the ISS-21 genome (26).

**FIG 3:**
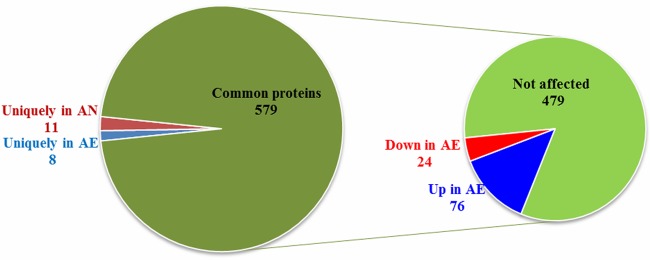
Distribution of *C. butyricum* type E strain ISS-190 proteins identified by proteomic analysis. Diagram showing the distributions of the cellular proteins identified in Clostridium butyricum strain ISS-190 uniquely in aerobiosis (AE), in anaerobiosis (AN), or under both conditions (common proteins). On the right, the diagram shows how many common proteins were found up- or downexpressed in AE (|FC| > 0.6, respectively; *P* values < 0.05) or were not affected by the oxygen concentration.

To identify differentially expressed proteins (DEPs) following air exposure, proteins from cells grown under AN or AE conditions were analyzed by proteomic analysis. A total of 953 proteins were detected; however, only 598 proteins were consistently identified in at least 3 of 5 replicates and were therefore selected for further analyses (see Table S1 in the supplemental material). Among them, 8 and 11 proteins were uniquely expressed in either the AE or AN environment, whereas 579 were identified in both environments and were subjected to quantitative analysis. Of these, 76 proteins were upregulated and 24 were downregulated under AE conditions compared to AN conditions (Fig. 3). The analysis of proteins modulated by air exposure indicated that protein-protein interactions were enriched (*P* value of 6.3e−10), suggesting that specific protein complexes and/or networks were likely affected by oxygen (Fig. S1).

10.1128/mSystems.00299-18.2 FIG S1 Protein-protein interaction (PPI) network of DEPs. PPI network of proteins found up- or downrepresented or uniquely identified under the AE or AN condition constructed and visualized by STRING. The PPI enrichment *P* value was 6.3e−10. Download FIG S1, TIF file, 2.0 MB. Copyright © 2019 Camerini et al. 2019 Camerini et al. This content is distributed under the terms of the Creative Commons Attribution 4.0 International license.

10.1128/mSystems.00299-18.1 TABLE S1 *C. butyricum* type E strain ISS-190 cell proteins identified under both AE and AN conditions or uniquely under the AE or AN condition. Download Table S1, DOCX file, 0.09 MB. Copyright © 2019 Camerini et al. 2019 Camerini et al. This content is distributed under the terms of the Creative Commons Attribution 4.0 International license.

**FIG 4:**
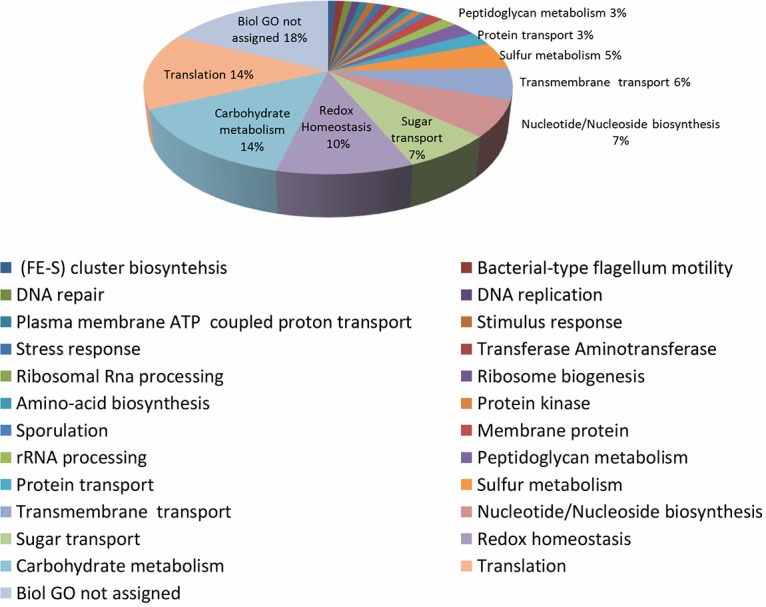
Functional categorization of DEPs under AE conditions. Functional categorization of the *C. butyricum* type E strain ISS-190 proteins modulated under AE conditions (*P* values < 0.05). The numbers in the graph indicate the percentage of the proteins involved in the described biological process.

**FIG 5:**
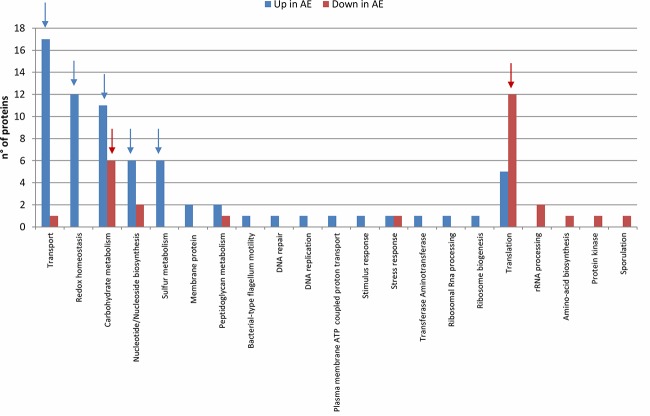
Frequency of DEPs under AE conditions in the different biological processes. Number of *C. butyricum* type E strain ISS-190 proteins up- or downmodulated under AE conditions (*P* values < 0.05) in each biological process. Arrows indicate the most-affected biological processes depending on AE/AN condition.

The DEPs were categorized by function (Table 1 and Fig. 4). The results indicate that the modulation of biological processes depends on the AE/AN growth conditions (Fig. 5). The most affected biological processes were membrane transport, redox homeostasis, carbohydrate metabolism, sulfur metabolism, and protein translation. The expression of ribosomal proteins was decreased under AE conditions. On the other hand, proteins involved in cell redox homeostasis, antioxidant defense, and sulfur metabolism and some proteins involved in the transport of solutes across membranes, particularly sugars and proteins, were overexpressed after air exposure. Similarly, several proteins involved in DNA damage responses and flagellum-associated proteins were more abundant under AE conditions.

**TABLE 1:** Clostridium butyricum ISS-190 proteins up- or downexpressed under the AE condition

Protein expression type, category, and no.	NCBI accession no.	Gene name	Protein name	FC^*a*^	Notes^*b*^	Biological process
Upexpressed						
Biological process not assigned						
1	489509402	CLP_2689	Phage major tail protein	1.89		Biol GO not assigned
2	489502155	CLP_3314	Metallophosphoesterase	0.69		Biol GO not assigned
3	488646122	CLP_3861	Outer surface protein	*		Biol GO not assigned
4	489502201	CLP_3345	Uncharacterized protein	1.55		Biol GO not assigned
5	489504250	CLP_1280	Uncharacterized protein	1.16		Biol GO not assigned
6	489504278	CLP_1290	Uncharacterized protein	1.28		Biol GO not assigned
7	489504289	CLP_1282	Uncharacterized protein	*		Biol GO not assigned
8	489504297	CLP_1288	Uncharacterized protein	1.31		Biol GO not assigned
9	489504751	CLP_2775	Putative lipoprotein	2.24		Biol GO not assigned
10	489503695	CLP_3097	Putative lipoprotein	0.70		Biol GO not assigned
11	489509462	CLP_1648	Major capsid protein	*		Biol GO not assigned
12	489504253	CLP_1279	Uncharacterized protein	1.54		Biol GO not assigned: likely phage
13	489504296	CLP_1287	Baseplate J family protein	1.29		Biol GO not assigned: likely phage
14	237656561	CLP_1654	Uncharacterized protein	*		Biol GO not assigned: likely PTS
Carbohydrate metabolism						
15	488642402	CLP_0032	*N*-Acetylneuraminate lyase (NanA)	2.89		Carbohydrate metabolism: sialic acid catabolism
16	489505355	CLP_0171	*N*-Acetylmannosamine-6- phosphate 2-epimerase (NanE)	4.50		Carbohydrate metabolism: sialic acid catabolism
17	489503203	CLP_0041	Beta-glucosidase	*	Glycoside hydrolase	Carbohydrate metabolism
18	489502642	CLP_0871	Alpha,alpha-phosphotrehalase	1.32	Glycoside hydrolase	Carbohydrate metabolism
19	489505662	CLP_1017	Alpha amylase, catalytic region	1.64	Glycoside hydrolase	Carbohydrate metabolism
20	489502184	CLP_3347	Glycosyl hydrolase family 32, N domain protein	3.15	Glycoside hydrolase	Carbohydrate metabolism
21	488644093	CLP_1327	Hydroxyacylglutathione hydrolase	1.57		Carbohydrate metabolism
22	488646082	CLP_3906	Ribose-5-phosphate isomerase B	3.45		Carbohydrate metabolism
23	489502147	CLP_3305	dTDP-4-dehydrorhamnose 3,5-epimerase	1.35		Carbohydrate metabolism
24	489503814	CLP_3464	UDP-*N*-acetylglucosamine 4,6-dehydratase	*		Carbohydrate metabolism
25	489507281	CLP_4271	Aldose 1-epimerase	0.65		Carbohydrate metabolism
Peptidoglycan metabolism						
26	489504484	CLP_3010	Penicillin-binding protein	2.12		Peptidoglycan metabolism
27	489502929	CLP_0014	Cell wall hydrolase/autolysin	4.04		Peptidoglycan metabolism
Redox homeostasis						
28	488644028	CLP_1392	FAD-dependent oxidoreductase	0.94	Fe-S	Redox homeostasis
29	488644495	CLP_1011	ArsC family protein	2.63		Redox homeostasis
30	488644556	CLP_0948	Redoxin	1.37		Redox homeostasis
31	489501807	CLP_2414	NADH oxidase	2.89	NADH→NAD	Redox homeostasis
32	489503213	CLP_0082	Oxidoreductase NAD-binding domain protein	2.16	NAD→NADH	Redox homeostasis
33	489505820	CLP_3877	FAD-linked oxidase domain protein	1.58	FAD→FADH	Redox homeostasis
34	489506590	CLP_0595	Thioredoxin-disulfide reductase	3.25	FAD→FADH, NAD→NADH	Redox homeostasis
35	489506753	CLP_0519	MviM protein NAD(P)-dependent oxidoreductase	3.25	NADP→NADPH	Redox homeostasis
36	489507464	CLP_4064	Thioredoxin reductase	1.74	NADP→NADPH	Redox homeostasis
37	489510856	CLP_0102	Rubrerythrin	1.45	Fe	Redox homeostasis
38	488644026	CLP_1394	Zinc finger protein	0.67		Redox homeostasis
39	489502510	CLP_2859	Ribosome-binding ATPase YchF	3.21		Redox homeostasis
Sulfur metabolism						
40	489504716	CLP_1763	Cystathionine beta-lyase, MetC	2.10		Sulfur metabolism: amino acid metabolism
41	489507701	CLP_3701	*S*-Adenosylmethionine synthase, MetK	1.02		Sulfur metabolism: Cys and Met metabolism
42	489505568	CLP_1012	Sulfite reductase	1.25	Fe-S	Sulfur metabolism: H_2_S biosynthesis—redox homeostasis
43	237656426	CLP_2257	Sulfate adenylyltransferase subunit 1-bifunctional enzyme CysN/CysC	2.93		Sulfur metabolism: H_2_S biosynthesis—sulfate reduction
44	489501666	CLP_2258	Sulfate adenylyltransferase subunit 2-bifunctional enzyme CysN/CysC	*		Sulfur metabolism: H_2_S biosynthesis—sulfate reduction
45	488642894	CLP_2741	Cysteine desulfurase IscS	1.18	Fe-S	Sulfur metabolism: (Fe-S) cluster biosynthesis
Transport						
Protein transport						
46	488644099	CLP_1321	Protein translocase subunit SecD	1.20	P-P hydrolase	Protein transport
47	489510437	CLP_1322	Protein-export membrane protein SecF	0.64	P-P hydrolase	Protein transport
48	489504061	CLP_3432	Chemotaxis protein MotA	0.69		Protein transport
Sugar transport						
49	488646019	CLP_3984	Phosphoenolpyruvate-protein phosphotransferase	1.19	Sugar PTS	Sugar transport
50	489503218	CLP_0055	PTS, lactose/cellobiose family IIB component	2.05	Sugar PTS	Sugar transport
51	489505845	CLP_3908	PTS enzyme IIC component, galactitol transporter	2.57	Sugar PTS	Sugar transport
52	489505963	CLP_3910	PTS enzyme IIBC component, fructose transporter	2.96	Sugar PTS	Sugar transport
53	489507776	CLP_3760	Lichenan-specific phosphotransferase enzyme IIA component, cellobiose transporter	3.21	Sugar PTS	Sugar transport
54	489508885	CLP_3296	CspC, *N*-acetylmuramoyl- l-alanine amidase family protein	0.88	Hydrolase	Sugar transport: peptidoglycan amidohydrolase
55	489501903	CLP_2323	Putative sugar-binding secreted protein	4.50	ABC transporter	Sugar transport: peptidoglycan amidohydrolase
Transmembrane transport						
56	489510211	CLP_2264	Sulfate transporter subunit, sulfate starvation-induced protein 2	1.86	ABC transporter	Transmembrane transport
57	489507313	CLP_4206	ABC transporter, ATP-binding protein	2.04	ABC transporter	Transmembrane transport
58	489502974	CLP_2166	Amino acid permease- associated region	3.44		Transmembrane transport
59	489504117	CLP_2003	Efflux transporter, RND family	3.09		Transmembrane transport
60	488644700	CLP_0371	Extracellular solute-binding protein family 3	0.91		Transmembrane transport
61	489506027	CLP_3956	Extracellular solute-binding protein, family 1	0.77		Transmembrane transport
62	489502966	CLP_2159	Spermidine/putrescine import ATP-binding protein PotA	0.86		Transmembrane transport
Membrane protein						
63	489505750	CLP_1031	ErfK/YbiS/YcfS/YnhG family protein	1.22		Membrane protein
64	488645963	CLP_4057	PilT protein domain protein	1.47		Membrane protein
65	489502814	CLP_1221	V-type ATP synthase subunit D	3.67		Plasma membrane ATP- coupled proton transport
Nucleotide/ nucleoside biosynthesis						
66	489510278	CLP_1207	Pseudouridine synthase, RluA family	0.86		Nucleoside synthesis
67	488642701	CLP_3018	Nucleoside diphosphate kinase (NDK)	2.09		Nucleotide biosynthesis
68	488644020	CLP_1400	Uridine kinase (UDK)	1.27		Pyrimidine biosynthesis
69	488646320	CLP_4393	Oxidoreductase FAD/NAD	1.46	Fe-S (oxidoreductase)	Pyrimidine biosynthesis
70	489502503	CLP_2848	Dihydroorotate dehydrogenase B [NAD(+)], PyrK	0.84	Fe-S (electron transfer)	Pyrimidine biosynthesis
71	488642810	CLP_2846	Orotate phosphoribosyltransferase, PyrE	0.26		Pyrimidine biosynthesis
Translation						
72	489504979	CLP_2730	GTP-binding protein TypA/BipA	2.16		Translation
73	489502964	CLP_2156	Helicase domain protein	3.82		Translation
74	488645666	CLP_3735	Peptide chain release factor 1	1.05		Translation
75	489506025	CLP_3829	Asparaginyl/glutamyl-tRNA amidotransferase GatC	0.92		Translation
76	489503907	CLP_3482	Flagellar assembly factor FliW	1.25		Translation
Other						
77	489503965	CLP_3434	Flagellar basal body protein	*		Bacterial-type flagellum motility
78	489505096	CLP_2731	RNase J	1.10		rRNA processing
79	488642929	CLP_2579	GTPase Der	1.98		Ribosome biogenesis
80	489509514	CLP_1938	Chemotaxis response regulator	1.04		Stimulus response
81	488644530	CLP_0974	UspA domain protein	1.89		Stress response
82	489502173	CLP_3317	DegT/DnrJ/EryC1/StrS aminotransferase	0.85		Transferase aminotransferase
83	489510263	CLP_2488	Protein RecA	0.79		DNA repair
84	489505855	CLP_3987	Ribonucleoside-diphosphate reductase	1.54	Oxidoreductase	DNA replication
Downexpressed						
Biological process not assigned						
1	488644185	CLP_1269	Uncharacterized protein	−0.64		Biol GO not assigned
2	489503664	CLP_3067	Uncharacterized protein	−3.20		Biol GO not assigned
3	489504941	CLP_2749	p-47 protein	−2.11		Biol GO not assigned
4	489505689	CLP_1082	Uncharacterized protein	*		Biol GO not assigned
5	489501399	CLP_1582	TPR repeat protein	*		Biol GO not assigned
6	489507297	CLP_4257	Uncharacterized protein	*		Biol GO not assigned
7	489510276	CLP_2354	Uncharacterized protein	−1.42		Biol GO not assigned
Carbohydrate metabolism						
8	489509381	CLP_1388	Propionate CoA-transferase	−2.21	Likely uses butyryl- CoA as the substrate	Carbohydrate metabolism
9	489502979	CLP_0779	Transaldolase	−1.55		Carbohydrate metabolism
10	488644944	CLP_0793	Pyruvate formate-lyase-activating enzyme	−1.85	Fe-S	Carbohydrate metabolism
11	489505871	CLP_3853	Butyryl-CoA dehydrogenase	−0.95		Carbohydrate metabolism: butyrate synthesis pathway
12	489506129	CLP_3852	Electron transfer flavoprotein, beta subunit	−1.13	Electron transfer	Carbohydrate metabolism: butyrate synthesis pathway
13	906848776	CLP_3850	3-Hydroxybutyryl-CoA dehydrogenase	−1.24		Carbohydrate metabolism: butyrate synthesis pathway
Translation						
14	488643005	CLP_2502	30S ribosomal protein S15	−0.78		Translation
15	488645977	CLP_4028	30S ribosomal protein S19	−1.16		Translation
16	488645980	CLP_4025	50S ribosomal protein L16	−0.72		Translation
17	488645984	CLP_4021	50S ribosomal protein L24	*		Translation
18	488645990	CLP_4015	30S ribosomal protein S5	−0.77		Translation
19	488645998	CLP_4006	30S ribosomal protein S13	−1.66		Translation
20	488646007	CLP_3997	50S ribosomal protein L13	−0.87		Translation
21	488646008	CLP_3996	30S ribosomal protein S9	−0.77		Translation
22	488646514	CLP_4041	50S ribosomal protein L10	−0.84		Translation
23	489502574	CLP_2872	50S ribosomal protein L25	−1.72		Translation
24	489503008	CLP_2520	Ribosome-recycling factor	*		Translation
25	488646138	CLP_3844	60-kDa chaperonin	−0.94	Protein refolding	Translation
rRNA processing						
26	488642944	CLP_2563	Probable dual-specificity RNA methyltransferase RlmN	*	Fe-S	rRNA processing
27	489508929	CLP_3179	rRNA small-subunit methyltransferase	*		rRNA processing
Nucleotide/ nucleoside biosynthesis						
28	489507369	CLP_4280	Ribose-phosphate pyrophosphokinase	*		Nucleotide biosynthesis
29	488642862	CLP_2788	5-(Carboxyamino)imidazole ribonucleotide mutase, PurE	*		Nucleotide biosynthesis
Other						
30	488642931	CLP_2577	Stage IV sporulation protein A	−3.09		Sporulation
31	653633918	AWN73_ 11055	Serine protease	−2.83		Protein kinase
32	488643856	CLP_1581	Heat shock protein Hsp20	−2.40		Stress response
33	489507712	CLP_3749	Peptidoglycan-binding LysM	−2.19		Peptidoglycan metabolism
34	488643009	CLP_2498	Aspartokinase	*		Amino acid biosynthesis
35	489504328	CLP_3792	*N*-Acetylgalactosamine permease IID component	*	PTS	Sugar transport

a FC is reported as log_2_ AE/AN ratio, as described in Materials and Methods. * indicates proteins for which FC has not been calculated because they have been detected only in aerobic or anaerobic conditions.

b Notes column reports some specific relevant functional protein features discussed in the text.

With respect to carbohydrate metabolism, some proteins related to polysaccharide catabolic processes were upregulated in the AE environment, whereas three enzymes responsible for converting acetoacetyl-CoA to butyryl-CoA (3-hydroxybutyryl-CoA dehydrogenase, acyl-CoA dehydrogenase, and electron transfer flavoprotein subunit beta) were downregulated in the AE environment. Of note, the pyruvate formate lyase (PFL)-activating protein was also decreased in the AE environment.

### Effects of air exposure on extracellular metabolism evaluated by ^1^H NMR spectroscopy.

**FIG 6:**
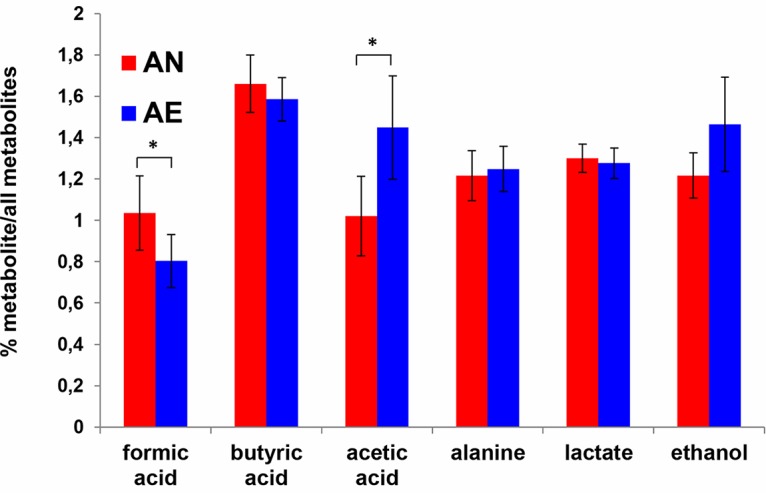
Extracellular metabolite concentrations from pooled ISS strains (*n* = 6). Relative concentrations (%) of the individual metabolites versus all extracellular metabolites investigated are shown on the *y* axis. *, *P* < 0.05 according to Student’s *t* test.

Nuclear magnetic resonance (NMR) spectroscopy was used to identify and quantify fermentation products in the extracellular medium for both strains after a 5-h incubation under either AN or AE conditions (Fig. 6).

The formate levels were significantly lower (*P* = 0.004) in the AE extracellular medium than in the AN extracellular medium, whereas the acetate content was significantly higher in the AE medium than in the AN medium (*P* = 0.003). Moreover, there was a significant increase in the acetate/butyrate ratio (*P* = 0.004) in the media of both strains under AE conditions compared to the strains grown under AN conditions. There were no other significant differences in the levels of metabolites linked to pyruvate metabolism (lactate, ethanol, and alanine).

## DISCUSSION

Anaerobically cultured *C. butyricum* type E strains continued to grow and produce BoNT/E in liquid medium during the transition from anaerobic to aerated conditions, although aeration for 5 h resulted in a dissolved oxygen concentration in the final cultures of ∼5 ppm, which does not support aerobic life (27) but is not the oxygen-free conditions required for vegetative growth of anaerobic clostridia.

While the OD_600_ data indicated similar growth rates under AN and AE conditions over 5 h, viable cell counts were lower in the AE environment than in the AN environment at the end of the culture period, suggesting that bacterial growth was decreased under the stress conditions of aeration compared to the ideal conditions of anaerobiosis. The lack of complete correlation between OD and viable cell count data under stress conditions has been reported for other bacteria (28) and may be because OD values are affected by light scattering due to cell debris and stress-induced bacterial cell damage.

*C. butyricum* type E strains continued to grow upon air exposure only when the starting exponentially growing cultures contained at least 10^3^ cells/ml. The absence of growth at lower initial bacterial densities could be a result of either oxygen toxicity or decreased cell-to-cell signaling (24). The absence of spores in the cultures of *C. butyricum* type E strains after a 5-h aeration indicates that sporulation did not occur in the evaluated period and that aerotolerance was not due to spore formation. Sporulation in clostridia has been reported to be strain dependent (29).

Of note, our results indicated that even BoNT/E production was maintained during the transition from AN to AE conditions. BoNT/E protein and toxicity levels were increased in the cultures transferred to AE conditions for 24 h, although the levels were significantly lower than those in cultures maintained under AN conditions for the same period. The significantly lower BoNT/E protein and toxicity levels detected in the AE environment than those in the AN environment can be attributed to the decreased growth rates under the former conditions. Moreover, our proteomics analysis revealed that the abundance of several ribosomal proteins was lower under AE conditions, evidencing that the overall protein synthesis could be downregulated in the presence of oxygen, which is a general adaptive response to acute stress in bacteria (30). Additional effects due to downregulation of BoNT/E synthesis under AE conditions cannot be excluded. Little is known on the toxin synthesis regulation in BoNT/E-producing clostridial strains. We recently hypothesized that production of BoNT/E in *C. butyricum* type E strains might be controlled at the posttranscriptional and/or posttranslational levels (i.e., protein folding, secretion, and degradation) (31); accordingly, since oxygen is known to modify the structure and function of proteins, BoNT/E inactivation by oxidative damage could be expected (32). Recently, it was shown that BoNT/E expression in C. botulinum type E strains is positively regulated by the sporulation regulator Spo0A (33); our proteomics results showed similar Spo0A protein levels in the *C. butyricum* type E broth cultures incubated for 5 h under AN and under AE conditions (see Table S1 in the supplemental material). The absence of significant differences in the BoNT/E protein levels under AN and AE conditions in the proteomics analysis may be due to only cellular proteins being analyzed whereas BoNT/E protein is an exotoxin secreted by clostridia in the medium (34). Nevertheless, cellular levels of the protein P-47 were decreased under AE conditions (Table 1); this protein is encoded in the *bont/e* toxin gene cluster and coexpressed with BoNT/E, although the two proteins might not be secreted together (35).

Our proteomics analyses indicated that membrane transport was one of the most affected processes under AE conditions: many proteins overexpressed in AE contain or interact with ATP-binding cassettes (ABC transporters) and are involved in transporting solutes across membranes. Other overexpressed proteins are involved in sugar transport, especially five proteins (Table 1) from the phosphoenolpyruvate (PEP)-dependent phosphotransferase system (PTS), which is a major mechanism used by bacteria for carbohydrate uptake and conversion into phosphoesters during transport (36). Moreover, the proteomic analysis indicated that acetylmuramoyl-l-alanine amidase and cell wall hydrolase, which are involved in peptidoglycan catabolism, were upregulated under AE conditions.

Other proteins involved in protein transport, including SecD and SecF, which are members of the Sec protein translocase complex, were overexpressed in the AE environment. These results indicate that molecular trafficking through the cellular membrane and carbohydrate metabolism are enhanced under AE conditions, suggesting that energy requirements are higher.

Aeration also induced the expression of proteins involved in redox homeostasis, including NADH oxidase, rubrerythrin, peroxiredoxin, and thioredoxin reductase. In this respect, Kawasaki et al. (4) detected the activity of NAD(P)H peroxidase and superoxide dismutase (SOD) in nonneurotoxigenic *C. butyricum* strains exposed to air. It is known that rubrerythrin has a strong NAD(P)H peroxidase activity (10). Moreover, enzymes homologous to peroxiredoxin and thioredoxin reductase prevent the inactivation of manganese-SOD (Mn-SOD) of Saccharomyces cerevisiae under oxidative stress, contributing to the antioxidant defense in yeast (37). Since an Mn-SOD gene is carried in the genome of three closely related neurotoxigenic *C. butyricum* type E strains, we speculate that peroxiredoxin and thioredoxin reductase in these strains may protect Mn-SOD.

**FIG 7:**
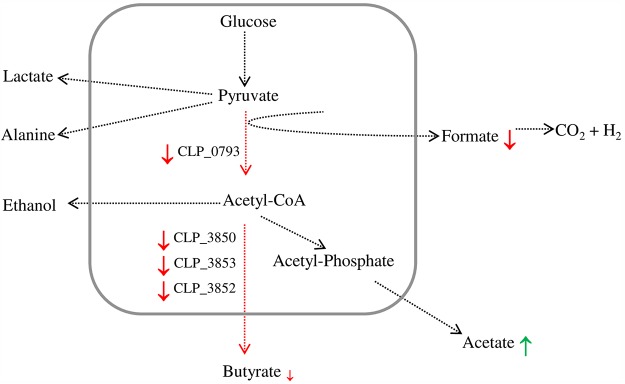
Fermentation pathways of glucose under AE and AN conditions detected by MS and NMR analyses. End products from pyruvate metabolism detected by NMR analysis from the extracellular medium are shown out of the box: red and green arrows indicate down- and upmodulation, respectively, under the AE condition. Enzymes involved in butyrate production and detected by proteomic analysis are shown inside the box with a red arrow indicating their downmodulation. (CLP_0793, pyruvate formate-lyase-activating enzyme; CLP_3850, 3-hydroxybutyryl-CoA dehydrogenase; CLP_3853, butyryl-CoA dehydrogenase; CLP_3852, electron transfer flavoprotein, beta subunit).

The proteomic analysis indicated that proteins involved in sulfur metabolism were upregulated under AE conditions, including two subunits of the bifunctional enzyme CysN/CysC sulfate adenylyltransferase and sulfite reductase: these enzymes are involved in cysteine production, and this sulfur-containing amino acid strongly inhibits toxin production in C. difficile (38). Furthermore, several cysteine-containing proteins, often required for protein folding or involved in cellular response to oxidative stress, and (Fe-S) cluster-containing proteins are sensitive to oxidization, and many of these proteins were overexpressed under AE conditions (Table 1), suggesting the need to restore the pool of these proteins in their active state. In contrast, the PFL-activating enzyme, an oxygen-sensitive Fe-S binding protein, responsible for the conversion of pyruvate into acetyl-CoA and formate in anaerobic metabolism (Fig. 7), was downregulated under AE conditions. In accordance, the formate content was significantly lower in the supernatants from aerated *C. butyricum* type E cells than in those from anaerobic cells. Formate is ultimately converted to carbon dioxide and hydrogen in the metabolic pathways of clostridial species (Fig. 7). Therefore, the lower levels of formate resulting from the decreased expression of the PFL-activating enzyme, together with the upregulation of hydrogen-consuming enzymes, may partially explain the decreased formation of gas in the cultures upon aeration.

Furthermore, the proteomics analysis showed that three enzymes of the butyrate metabolic pathway were downregulated under AE conditions, suggesting that oxygen induced a decrease in butyrate biosynthesis. In line with this result, butyrate content was decreased, although not significantly, in the aerated supernatants compared to nonaerated supernatants; concomitantly, the acetate levels were significantly higher in the aerated supernatants, suggesting that oxygen caused a shift in electron flow toward acetate formation instead of butyrate formation in these *C. butyricum* type E strains. The acetate/butyrate ratio in nonneurotoxigenic *C. butyricum* strains is increased as the hydrogen partial pressure is decreased in the medium (39). Moreover, the higher levels of several proteins putatively involved in DNA replication and repair and nucleotide biosynthesis and flagellum-related proteins under AE conditions suggest that the DNA damage response may be activated and proton-driven bacterial motility enhanced. The improved bacterial motility may increase proton consumption and consequently decrease the generation of ROS (40). Furthermore, our finding that a protein involved in an early stage of sporulation was down-expressed in the AE environment is consistent with the absence of spores in *C. butyricum* type E cultures after a 5-h exposure to ambient air.

In conclusion, the enhanced aerotolerance of neurotoxigenic *C. butyricum* type E strains that we report here may have public health significance. First, it may increase the opportunities for these microorganisms to colonize the newborn intestine, because the intestine at birth is aerobic and gradually becomes anaerobic (18); in addition, the ability to inactivate the ROS generated by inflammatory processes in the gut may be advantageous to intestine-colonizing neurotoxigenic *C. butyricum* type E strains. To date, most neurotoxigenic *C. butyricum* type E strains have been involved in infant intestinal toxemia botulism (21–23, 41, 42). It is of interest that Cassir et al. (43) recently found a significant association between oxidized gut environment and the presence of cytotoxic (nonneurotoxigenic) *C. butyricum* strains in preterm neonates with necrotizing enterocolitis. Furthermore, the studies on aerotolerance may improve the isolation and identification of BoNT-producing clostridia, considering that other clostridial strains have been misidentified because of their aerotolerance features (11).

To our knowledge, this study is the first to demonstrate vegetative growth and toxin production upon air exposure for BoNT-producing clostridial strains. The results point to the need for further research on the aerotolerance of other BoNT-producing clostridial strains, especially C. botulinum strains, which are more frequently involved in botulism.

## MATERIALS AND METHODS

### Bacterial strains and growth conditions.

Two neurotoxigenic *C. butyricum* type E strains (ISS-21 and ISS-190) isolated from distinct infant botulism cases in Italy were used in this study (41, 42). The effect of atmospheric oxygen on the vegetative growth of these strains was analyzed by using a culture approach similar to that used by other authors for similar purposes (6, 14, 15), with modifications. Tryptone-peptone-glucose-yeast extract (TPGY) broth (pH 7.0) (Oxoid) without the addition of reducing agents was used to grow the strains. Clostridial growth was monitored by measuring the optical densities of the cell suspensions at 600 nm (OD_600_) at regular time intervals (Biophotometer; Eppendorf). The strains were grown to the mid-exponential phase (OD_600_ of ∼1) at 37°C in a jar with an anaerobic gas generator (Anaerogen; Oxoid) (starting cultures). Then, 50% of the exponentially growing cultures were transferred to flasks (flask-to-medium ratio, 1:10) and incubated in ambient air (aerated or AE conditions) in an incubator shaker (New Brunswick Scientific; model G25) at 200 rpm and 37°C for 5 h; the remaining volumes of the starting cultures were overlaid with sterile Vaseline oil to produce oxygen-depleted (anaerobic or AN) conditions and were incubated at 37°C for 5 h. The experimental time of 5 h was empirically chosen in order to assay the differences between the vegetative cultures under the AN and AE conditions before sporulation occurred. Growth curves were generated using the OD_600_ values. These experiments were repeated three times.

The AE cultures were streaked on nonselective tryptone soy agar (TSA) plates, and the plates were incubated at 37°C for 24 h to detect contamination with aerobic bacteria at the end of culturing. To assess the presence of spores, slides from the AE cultures were examined microscopically; in addition, aliquots of the AE cultures grown for 5 h were treated at 70°C for 10 min and streaked on egg yolk agar plates, and bacterial growth was assessed after a 48-h incubation at 37°C under anaerobiosis. The pH values of the cultures were determined using a pH meter (Mettler Toledo). The dissolved oxygen levels were measured using a dissolved oxygen meter (model HI9146; Hanna Instruments). Viable bacteria were counted using the three-tube most probable number (MPN) method as previously described (31). The average number of viable cells was measured by combining the data from three separate experiments.

Student’s *t* test was used to perform all pairwise comparisons between OD_600_ values or viable cell counts under AN and AE conditions. All calculations were performed using GraphPad Prism software version 6 (GraphPad Software, San Diego, CA), and *P* values < 0.05 were considered statistically significant.

### Quantification of BoNT/E protein and toxicity levels.

BoNT/E protein and toxicity levels were measured in the starting (mid-exponential-growth-phase) cultures and in cultures grown under AN or AE conditions for 24 h. The cultures were centrifuged at 12,000 × *g* for 20 min at 4°C.

BoNT/E protein levels in the supernatants from two assays were quantified in duplicate using a commercial BoNT/E enzyme-linked immunosorbent assay (ELISA) kit (Tetracore). Absorbance was measured at 405 nm using a Multiskan Go microplate spectrophotometer (Thermo Scientific). The BoNT/E concentrations (ng/ml) were determined using the standard toxin concentration curve provided by the manufacturer, and the mean and standard deviation were calculated. Student’s *t* test was used to perform pairwise comparisons, with statistical significance at *P* < 0.05.

A mouse bioassay was used for determining BoNT/E toxicity. The culture supernatants were treated with 0.5% trypsin at 1:250 for 20 min at room temperature, 2-fold diluted in phosphate buffer (pH 6.4) containing 0.2% gelatin, and injected intraperitoneally into groups of two male CD1 mice (weight of 25 g). The mice were monitored over 4 days for signs of botulism (44), the number of deaths was recorded, and the results were expressed as mouse lethal dose (MLD) per ml. The mouse bioassay was done twice.

The experimental animal protocol adhered to Directive 2010/63/EU on the protection of animals used for scientific purposes of the European Parliament and was approved by the Italian Ministry of Health (authorization no. 291/2015).

### Harvesting and lysis of bacterial cells.

Fifty milliliters of the AE and AN cultures grown for 5 h was centrifuged at 5,000 × *g* for 15 min. Each culture supernatant (2 ml) was mixed with 18 ml of 80% ethanol and stored at −80°C until analysis of metabolites. For cell protein extraction, the pellets were washed twice with 50 mM phosphate buffer (pH 7.8) containing 0.1 mM EDTA and suspended in 500 μl of a solution containing 25 mM Tris, pH 7.4, 0.1 mM EDTA, 0.2% Triton X-100, 0.2 mg/ml lysozyme, 0.1 mg/ml RNase, 0.1 mg/ml DNase, and 1× protease inhibitor cocktail (Sigma-Aldrich). After 30 min at 37°C, the suspensions were subjected to ultrasound treatment (10 cycles of 45 s on and 2 min off at a frequency of 20 kHz) (Sonics VCX 500) on ice. Samples were centrifuged at 25,000 × *g* for 10 min at 4°C, and the protein concentration in the supernatants was estimated using the Bio-Rad protein assay kit with bovine serum albumin used as a standard. The supernatants were stored at −80°C until proteomic analyses.

### Proteomic analysis.

Proteins were separated on a 1-D gel, 4 to 12%, and colored by Coomassie blue staining. Each gel lane was cut in 12 to 15 contiguous slices, treated with DTT and iodoacetamide, and digested with trypsin (Promega Corporation), as described by Shevchenko et al. (45). The peptide mixtures were analyzed by nanoflow reversed-phase liquid chromatography–tandem mass spectrometry (nLC-MS/MS) using an Ultimate 3000 HPLC (Dionex) coupled online with a linear ion trap (LTQ; Thermo). Peptides were desalted in a trap column and separated in a 10-cm silica capillary as previously described (46). Tandem mass spectra were acquired in data-dependent mode (top five) and searched against *Clostridium* proteins (*C. butyricum* type E strain BL5262 taxonomy identity 632245) from the NCBI database using Proteome Discoverer version 1.4 (Thermo Electron) filtering spectral matches with a Percolator node with a *q*-value based false-discovery rate (FDR) of 0.01. Specific trypsin cleavages were admitted, with two possible missed cleavages. Only proteins identified with at least two peptides were considered, and only proteins detected in at least three replicates were considered for further analysis. The analyses were performed in quintuplicates. A label-free quantitative analysis was performed to identify DEPs in proteomic analyses, comparing AE and AN conditions. Only proteins identified in at least three replicates were selected for identification of DEPs. The proteins expressed under one condition were excluded from the statistical analysis but used for the successive analysis step. Protein abundances were estimated using normalized spectral abundance factor (NSAF) values calculated normalizing the spectral counts against the protein length and the sum of spectral counts in the corresponding run. Identification of DEPs was performed using the method proposed by Pavelka et al. (47), effectively applied in spectral-count-based proteome quantification (48), showing a good sensitivity-specificity tradeoff. The method consists of signal-to-noise-ratio (STN) statistics improved using protein-specific estimates of standard deviation (SD) derived by the power-law global error model (PLGEM), which well describes the SD-versus-mean dependence in NSAF data sets. A resampling-based algorithm is then used for multiple testing adjustment to control the false-positive rate (FPR). The PLGEM-STN method was applied using the ‘plgem’ Bioconductor (49) package in step-by-step mode. Further details on the method are reported in the work of Pavelka et al. (47). Our NSAF data set showed a good fitting to the PLGEM (adjusted *r*^2^ = 0.996); PLGEM-STN was thus used to compare NSAF values under AE and AN conditions; multiple testing correction was applied, setting in ‘plgem’ a 1,000-iteration resampling and a ‘delta’ (parameter for FPR estimate) of 0.05. The fold change (FC) for each protein was estimated as the log_2_ of the average NSAF(AE)/average NSAF(AN) ratio. The proteins showing both adjusted *P* values of <0.05 and |FC| of >0.6 were predicted as differentially expressed in our data set. The proteins up- or downrepresented and those uniquely identified under AE or AN conditions were grouped by biological function as retrieved from the UniProt database and analyzed by the STRING tool, version 10.5 (http://www.string-db.org), with a minimum required interaction score of 0.7.

### High-resolution NMR spectroscopy.

High-resolution NMR (Bruker Avance spectrometer; Karlsruhe, Germany) analyses were performed on extracellular medium at 25°C and 400 MHz. ^1^H magnetic resonance spectroscopy (MRS) spectra were acquired and analyzed as previously described (50). Metabolite quantification was expressed as the percentage of metabolite concentration versus all investigated metabolites. Differences in the metabolite concentration under AE and AN conditions were evaluated using the Wilcoxon signed-rank test at a significance level of *P* < 0.01.

### Availability of data.

The mass spectrometry proteomics data have been deposited in the ProteomeXchange Consortium (http://proteomecentral.proteomexchange.org) via the PRIDE partner repository with the data set identifier PXD013383.
