# Supplementary figures and images for: Type E Botulinum Neurotoxin-Producing Clostridium butyricum Strains Are Aerotolerant during Vegetative Growth
Source: mSystems. 2019 Apr 30;4(2):e00299-18. doi: 10.1128/mSystems.00299-18 (PMC6495232; doi:10.1128/mSystems.00299-18)

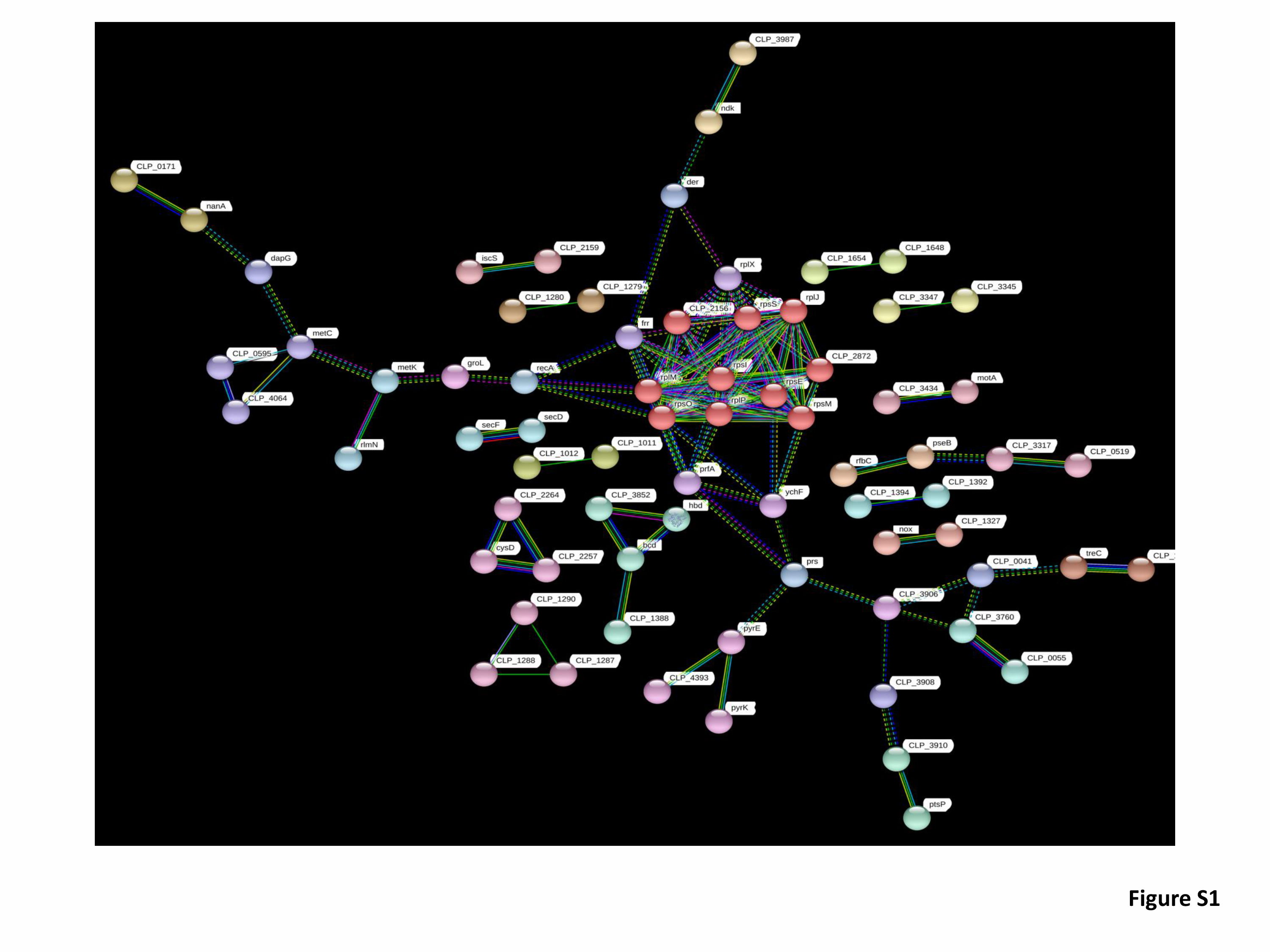

Supplement: FIG S1 [file mSystems.00299-18-sf001.tif]
