# Supplementary material for: Type E Botulinum Neurotoxin-Producing Clostridium butyricum Strains Are Aerotolerant during Vegetative Growth
Source: mSystems. 2019 Apr 30;4(2):e00299-18. doi: 10.1128/mSystems.00299-18 (PMC6495232; doi:10.1128/mSystems.00299-18)
Supplement: TABLE S1 [file mSystems.00299-18-st001.docx]

**Table_S1. *C. butyricum* type E strain ISS-190 cell proteins identified in both AE and AN conditions or uniquely in AE or AN conditions.**

|  | **Accession** | **Description** | **# AAs** | **MW** [kDa] | **AE** | **AN** |
| --- | --- | --- | --- | --- | --- | --- |
| ***Proteins identified in both AE and AN conditions*** | | | | | | |
| 1 | 237655040 | 1,4-alpha-glucan branching enzyme | 858 | 98.7 | 4 | 4 |
| 2 | 237656426 | bifunctional enzyme CysN/cysC | 557 | 63.1 | 3 | 2 |
| 3 | 237656562 | beta-galactosidase (Lactase) | 1008 | 117.3 | 1 | 3 |
| 4 | 237657891 | carbonate dehydratase | 183 | 20.8 | 2 | 3 |
| 5 | 237658260 | hypothetical protein CLP_3311 | 580 | 67.9 | 5 | 4 |
| 6 | 488642402 | N-acetylneuraminate lyase | 295 | 33.1 | 5 | 5 |
| 7 | 488642492 | HesB-like selenoprotein | 109 | 11.9 | 4 | 4 |
| 8 | 488642607 | [FeFe] hydrogenase H-cluster radical SAM maturase HydG | 472 | 53.7 | 5 | 5 |
| 9 | 488642610 | UDP-glucose 4-epimerase GalE | 329 | 36.1 | 5 | 5 |
| 10 | 488642696 | ATPase | 188 | 22.0 | 5 | 4 |
| 11 | 488642701 | nucleoside-diphosphate kinase | 137 | 15.5 | 5 | 3 |
| 12 | 488642715 | YigZ family protein | 215 | 24.2 | 2 | 3 |
| 13 | 488642777 | peptidase U32 | 786 | 89.4 | 3 | 1 |
| 14 | 488642803 | serine hydrolase | 258 | 29.1 | 5 | 5 |
| 15 | 488642810 | orotate phosphoribosyltransferase | 224 | 25.0 | 3 | 5 |
| 16 | 488642815 | aspartate--ammonia ligase | 337 | 38.8 | 3 | 4 |
| 17 | 488642821 | arginine--tRNA ligase | 562 | 63.5 | 5 | 5 |
| 18 | 488642825 | glutamate--tRNA ligase | 485 | 55.8 | 5 | 5 |
| 19 | 488642838 | hypothetical protein | 100 | 11.7 | 4 | 4 |
| 20 | 488642840 | hypothetical protein | 139 | 16.4 | 2 | 3 |
| 21 | 488642850 | paraslipin | 314 | 35.0 | 5 | 5 |
| 22 | 488642856 | cytochrome c551 | 55 | 6.6 | 3 | 3 |
| 23 | 488642876 | MarR family transcriptional regulator | 148 | 17.1 | 5 | 4 |
| 24 | 488642877 | ketoacyl-ACP synthase III | 323 | 35.1 | 5 | 5 |
| 25 | 488642879 | 2-nitropropane dioxygenase | 313 | 33.3 | 4 | 4 |
| 26 | 488642880 | malonyl CoA-acyl carrier protein transacylase | 312 | 34.2 | 3 | 2 |
| 27 | 488642881 | beta-ketoacyl-ACP reductase | 246 | 26.2 | 5 | 5 |
| 28 | 488642882 | beta-ketoacyl-[acyl-carrier-protein] synthase II | 410 | 43.4 | 5 | 5 |
| 29 | 488642884 | beta-hydroxyacyl-ACP dehydratase | 142 | 15.7 | 1 | 3 |
| 30 | 488642886 | acetyl-CoA carboxylase subunit beta | 297 | 33.5 | 4 | 3 |
| 31 | 488642887 | acetyl-CoA carboxylase carboxyltransferase subunit alpha | 272 | 30.3 | 2 | 2 |
| 32 | 488642894 | cysteine desulfurase NifS | 393 | 43.3 | 5 | 4 |
| 33 | 488642902 | hypothetical protein | 92 | 10.8 | 2 | 4 |
| 34 | 488642913 | cell division protein FtsZ | 377 | 40.1 | 5 | 5 |
| 35 | 488642929 | ribosome biogenesis GTPase Der | 438 | 49.8 | 3 | 2 |
| 36 | 488642930 | glycerol-3-phosphate dehydrogenase | 330 | 36.2 | 2 | 3 |
| 37 | 488642931 | stage IV sporulation protein A | 492 | 55.7 | 1 | 3 |
| 38 | 488642935 | hypothetical protein | 293 | 33.7 | 2 | 4 |
| 39 | 488642936 | hypothetical protein | 89 | 9.8 | 3 | 3 |
| 40 | 488642937 | guanylate kinase | 212 | 23.9 | 1 | 3 |
| 41 | 488642941 | peptide deformylase | 146 | 16.6 | 2 | 3 |
| 42 | 488642948 | ribulose-phosphate 3-epimerase | 214 | 23.0 | 3 | 4 |
| 43 | 488642951 | alkaline-shock protein | 116 | 12.8 | 4 | 4 |
| 44 | 488642955 | DNA methyltransferase | 185 | 20.9 | 2 | 2 |
| 45 | 488642956 | pantetheine-phosphate adenylyltransferase | 159 | 17.8 | 2 | 3 |
| 46 | 488642957 | hypothetical protein | 180 | 20.8 | 3 | 4 |
| 47 | 488642960 | phosphate acetyltransferase | 333 | 36.0 | 5 | 5 |
| 48 | 488642964 | phosphate acyltransferase | 333 | 36.3 | 3 | 3 |
| 49 | 488642966 | ribonuclease III | 232 | 26.6 | 2 | 3 |
| 50 | 488642969 | signal recognition particle-docking protein FtsY | 302 | 33.3 | 3 | 2 |
| 51 | 488642972 | 30S ribosomal protein S16 | 81 | 9.3 | 4 | 4 |
| 52 | 488642976 | 50S ribosomal protein L19 | 116 | 13.3 | 3 | 4 |
| 53 | 488642977 | ribosome biogenesis GTPase YlqF | 293 | 33.1 | 3 | 5 |
| 54 | 488642995 | ribosome maturation factor | 153 | 17.6 | 3 | 5 |
| 55 | 488642999 | translation initiation factor IF-2 | 697 | 76.1 | 5 | 5 |
| 56 | 488643001 | ribosome-binding factor A | 117 | 13.5 | 1 | 3 |
| 57 | 488643005 | 30S ribosomal protein S15 | 87 | 10.1 | 4 | 4 |
| 58 | 488643017 | ribosomal protein S12 methylthiotransferase RimO | 464 | 53.2 | 3 | 4 |
| 59 | 488643021 | stage V sporulation protein S | 86 | 8.9 | 2 | 3 |
| 60 | 488643022 | PTS sugar transporter subunit IIA | 86 | 9.1 | 4 | 4 |
| 61 | 488643024 | adenylosuccinate lyase | 476 | 54.0 | 5 | 5 |
| 62 | 488643106 | single-stranded DNA-binding protein | 220 | 25.2 | 5 | 5 |
| 63 | 488643108 | 2,3,4,5-tetrahydropyridine-2,6-dicarboxylate N-acetyltransferase | 236 | 25.1 | 2 | 5 |
| 64 | 488643109 | aspartate aminotransferase | 380 | 43.1 | 3 | 4 |
| 65 | 488643126 | molecular chaperone Hsp33 | 293 | 31.9 | 5 | 5 |
| 66 | 488643157 | PFL family protein | 451 | 47.4 | 2 | 3 |
| 67 | 488643192 | NADPH-dependent 7-cyano-7-deazaguanine reductase QueF | 167 | 19.6 | 2 | 3 |
| 68 | 488643263 | 2-hydroxy-acid oxidase | 477 | 51.5 | 3 | 3 |
| 69 | 488643351 | phosphoribosylpyrophosphate synthetase | 363 | 41.3 | 3 | 5 |
| 70 | 488643374 | hypothetical protein | 105 | 11.9 | 4 | 4 |
| 71 | 488643380 | N-acetylmannosamine kinase | 298 | 33.1 | 3 | 1 |
| 72 | 488643445 | 4-hydroxy-3-methylbut-2-enyl diphosphate reductase | 634 | 71.0 | 5 | 5 |
| 73 | 488643495 | fructose-1,6-bisphosphate aldolase, class II | 287 | 31.0 | 5 | 5 |
| 74 | 488643535 | chemotaxis protein CheX | 151 | 16.2 | 2 | 3 |
| 75 | 488643738 | chemotaxis protein CheV | 304 | 34.2 | 5 | 5 |
| 76 | 488643856 | heat-shock protein Hsp20 | 148 | 17.1 | 2 | 5 |
| 77 | 488643889 | hypothetical protein | 268 | 30.6 | 1 | 5 |
| 78 | 488643907 | arginine repressor | 150 | 16.4 | 3 | 3 |
| 79 | 488643915 | N utilization substance protein B | 131 | 15.0 | 4 | 4 |
| 80 | 488643916 | hypothetical protein | 126 | 13.5 | 4 | 3 |
| 81 | 488643926 | elongation factor P | 185 | 20.9 | 5 | 5 |
| 82 | 488643941 | bifunctional pyr operon transcriptional regulator/uracil phosphoribosyltransferase | 182 | 20.4 | 2 | 5 |
| 83 | 488643946 | cell division protein SepF | 152 | 17.3 | 4 | 5 |
| 84 | 488643947 | YggS family pyridoxal phosphate enzyme | 221 | 25.7 | 4 | 5 |
| 85 | 488643959 | 16S rRNA (cytosine(1402)-N(4))-methyltransferase | 310 | 35.3 | 3 | 2 |
| 86 | 488643962 | GTP-binding protein | 595 | 68.8 | 4 | 4 |
| 87 | 488643973 | hemerythrin | 131 | 15.8 | 3 | 5 |
| 88 | 488643995 | serine hydroxymethyltransferase | 410 | 45.1 | 5 | 5 |
| 89 | 488644020 | uridine kinase | 205 | 23.6 | 4 | 4 |
| 90 | 488644026 | molybdopterin oxidoreductase | 117 | 12.6 | 4 | 5 |
| 91 | 488644028 | FAD/NAD(P)-binding oxidoreductase | 476 | 52.3 | 5 | 5 |
| 92 | 488644033 | aldolase | 205 | 23.4 | 4 | 4 |
| 93 | 488644090 | aspartate--tRNA ligase | 599 | 68.0 | 5 | 5 |
| 94 | 488644091 | histidine--tRNA ligase | 419 | 47.7 | 4 | 5 |
| 95 | 488644093 | MBL fold hydrolase | 199 | 21.6 | 2 | 2 |
| 96 | 488644096 | adenine phosphoribosyltransferase | 172 | 18.6 | 5 | 5 |
| 97 | 488644099 | protein-export membrane protein SecD | 419 | 44.5 | 3 | 3 |
| 98 | 488644185 | hypothetical protein | 333 | 35.5 | 5 | 5 |
| 99 | 488644186 | nitrogen-fixing protein NifU | 230 | 24.6 | 5 | 5 |
| 100 | 488644240 | V-type ATP synthase subunit B | 459 | 51.0 | 5 | 5 |
| 101 | 488644241 | ATP synthase subunit A | 592 | 64.9 | 5 | 5 |
| 102 | 488644243 | ATP synthase subunit C | 333 | 38.4 | 5 | 5 |
| 103 | 488644245 | V-type ATP synthase subunit K | 164 | 16.7 | 3 | 3 |
| 104 | 488644246 | ATP synthase subunit I | 651 | 72.8 | 5 | 5 |
| 105 | 488644265 | hypothetical protein | 108 | 12.2 | 4 | 4 |
| 106 | 488644275 | ATP-dependent Clp endopeptidase, proteolytic subunit ClpP | 201 | 22.1 | 3 | 4 |
| 107 | 488644276 | trigger factor | 427 | 48.3 | 5 | 5 |
| 108 | 488644374 | hypothetical protein | 285 | 31.6 | 2 | 5 |
| 109 | 488644410 | fructose-6-phosphate aldolase | 214 | 23.2 | 5 | 5 |
| 110 | 488644495 | ArsC family transcriptional regulator | 115 | 13.2 | 4 | 2 |
| 111 | 488644502 | purine-nucleoside phosphorylase | 237 | 26.0 | 4 | 4 |
| 112 | 488644509 | aminotransferase | 392 | 43.9 | 2 | 2 |
| 113 | 488644530 | universal stress protein | 144 | 16.3 | 3 | 2 |
| 114 | 488644544 | 2-deoxyribose-5-phosphate aldolase | 220 | 23.9 | 4 | 2 |
| 115 | 488644556 | peroxiredoxin | 157 | 18.1 | 5 | 4 |
| 116 | 488644700 | amino acid ABC transporter substrate-binding protein | 286 | 31.1 | 5 | 5 |
| 117 | 488644786 | hypothetical protein | 305 | 34.4 | 2 | 4 |
| 118 | 488644874 | glucosamine-6-phosphate deaminase | 251 | 27.7 | 3 | 1 |
| 119 | 488644944 | pyruvate formate lyase-activating protein | 264 | 30.3 | 3 | 5 |
| 120 | 488645073 | hypothetical protein | 124 | 14.1 | 3 | 2 |
| 121 | 488645074 | PTS mannose transporter subunit IID | 303 | 33.1 | 5 | 5 |
| 122 | 488645076 | PTS mannose transporter subunit EIIAB | 325 | 35.3 | 5 | 5 |
| 123 | 488645138 | desulfoferrodoxin | 126 | 14.0 | 4 | 4 |
| 124 | 488645292 | RNA polymerase sigma factor RpoD | 369 | 42.2 | 5 | 5 |
| 125 | 488645306 | aspartyl-tRNA amidotransferase subunit B | 150 | 17.0 | 5 | 5 |
| 126 | 488645308 | histidine triad nucleotide-binding protein | 114 | 12.7 | 4 | 4 |
| 127 | 488645315 | molecular chaperone DnaJ | 377 | 40.5 | 3 | 4 |
| 128 | 488645316 | molecular chaperone DnaK | 613 | 65.6 | 5 | 5 |
| 129 | 488645317 | nucleotide exchange factor GrpE | 201 | 23.1 | 5 | 5 |
| 130 | 488645324 | 30S ribosomal protein S20 | 87 | 9.5 | 1 | 3 |
| 131 | 488645331 | DNA-directed RNA polymerase sigma-70 factor | 252 | 29.2 | 3 | 4 |
| 132 | 488645332 | anti-sigma F factor | 143 | 15.9 | 5 | 5 |
| 133 | 488645333 | anti-sigma F factor antagonist | 111 | 12.6 | 3 | 4 |
| 134 | 488645391 | response regulator | 119 | 13.2 | 4 | 4 |
| 135 | 488645403 | pyruvate:ferredoxin (flavodoxin) oxidoreductase | 1169 | 127.0 | 5 | 5 |
| 136 | 488645411 | glutamate dehydrogenase | 448 | 48.9 | 4 | 5 |
| 137 | 488645415 | hypothetical protein | 133 | 15.7 | 4 | 3 |
| 138 | 488645465 | SsrA-binding protein | 156 | 18.2 | 2 | 2 |
| 139 | 488645474 | 2,3-bisphosphoglycerate-independent phosphoglycerate mutase | 512 | 56.0 | 5 | 5 |
| 140 | 488645477 | type I glyceraldehyde-3-phosphate dehydrogenase | 333 | 35.6 | 5 | 5 |
| 141 | 488645496 | lipid kinase | 296 | 33.0 | 2 | 5 |
| 142 | 488645578 | GTPase CgtA | 429 | 47.4 | 4 | 3 |
| 143 | 488645590 | septum site-determining protein MinD | 266 | 29.0 | 3 | 4 |
| 144 | 488645591 | septum site-determining protein MinC | 219 | 24.5 | 2 | 2 |
| 145 | 488645600 | rod shape-determining protein | 339 | 36.3 | 5 | 5 |
| 146 | 488645624 | tRNA (adenosine(37)-N6)-threonylcarbamoyltransferase complex dimerization subunit type 1 TsaB | 244 | 27.0 | 5 | 5 |
| 147 | 488645650 | ATP synthase F1 subunit epsilon | 133 | 15.1 | 4 | 4 |
| 148 | 488645651 | ATP synthase subunit beta | 463 | 50.3 | 5 | 5 |
| 149 | 488645652 | F0F1 ATP synthase subunit gamma | 282 | 31.1 | 3 | 5 |
| 150 | 488645657 | F0F1 ATP synthase subunit A | 226 | 25.1 | 2 | 3 |
| 151 | 488645659 | acetyl-CoA acetyltransferase | 393 | 41.4 | 5 | 5 |
| 152 | 488645662 | uracil phosphoribosyltransferase | 209 | 22.9 | 5 | 5 |
| 153 | 488645666 | peptide chain release factor 1 | 360 | 41.1 | 5 | 5 |
| 154 | 488645672 | CTP synthetase | 535 | 59.9 | 5 | 5 |
| 155 | 488645674 | hypothetical protein | 136 | 15.2 | 3 | 2 |
| 156 | 488645700 | hypothetical protein | 459 | 49.3 | 1 | 3 |
| 157 | 488645713 | peptide chain release factor 3 | 531 | 60.3 | 5 | 5 |
| 158 | 488645828 | copper ion-binding protein | 68 | 7.4 | 4 | 4 |
| 159 | 488645862 | pyruvate kinase | 473 | 50.4 | 5 | 5 |
| 160 | 488645870 | stress protein | 94 | 10.8 | 2 | 4 |
| 161 | 488645886 | transketolase | 314 | 33.4 | 3 | 3 |
| 162 | 488645899 | hexapeptide transferase | 191 | 20.7 | 2 | 5 |
| 163 | 488645903 | AbrB family transcriptional regulator | 80 | 9.3 | 4 | 4 |
| 164 | 488645963 | PIN/TRAM domain-containing protein | 371 | 41.0 | 3 | 3 |
| 165 | 488645971 | elongation factor Tu | 397 | 43.5 | 5 | 5 |
| 166 | 488645972 | 30S ribosomal protein S10 | 102 | 11.5 | 3 | 3 |
| 167 | 488645974 | 50S ribosomal protein L4 | 206 | 22.7 | 5 | 5 |
| 168 | 488645975 | 50S ribosomal protein L23 | 98 | 11.0 | 3 | 4 |
| 169 | 488645976 | 50S ribosomal protein L2 | 276 | 30.2 | 5 | 5 |
| 170 | 488645977 | 30S ribosomal protein S19 | 90 | 10.3 | 3 | 4 |
| 171 | 488645978 | 50S ribosomal protein L22 | 111 | 12.3 | 4 | 4 |
| 172 | 488645979 | 30S ribosomal protein S3 | 222 | 24.5 | 5 | 5 |
| 173 | 488645980 | 50S ribosomal protein L16 | 144 | 16.3 | 5 | 5 |
| 174 | 488645983 | 50S ribosomal protein L14 | 122 | 13.2 | 5 | 5 |
| 175 | 488645985 | 50S ribosomal protein L5 | 179 | 20.3 | 5 | 5 |
| 176 | 488645987 | 30S ribosomal protein S8 | 132 | 14.7 | 5 | 5 |
| 177 | 488645988 | 50S ribosomal protein L6 | 180 | 19.8 | 5 | 5 |
| 178 | 488645989 | 50S ribosomal protein L18 | 119 | 13.1 | 3 | 4 |
| 179 | 488645990 | 30S ribosomal protein S5 | 165 | 17.3 | 5 | 5 |
| 180 | 488645992 | 50S ribosomal protein L15 | 146 | 15.7 | 5 | 5 |
| 181 | 488645994 | adenylate kinase | 215 | 23.8 | 5 | 5 |
| 182 | 488645998 | 30S ribosomal protein S13 | 122 | 13.8 | 3 | 4 |
| 183 | 488645999 | 30S ribosomal protein S11 | 131 | 14.0 | 4 | 5 |
| 184 | 488646000 | 30S ribosomal protein S4 | 206 | 23.4 | 5 | 5 |
| 185 | 488646001 | DNA-directed RNA polymerase subunit alpha | 315 | 35.1 | 5 | 5 |
| 186 | 488646002 | 50S ribosomal protein L17 | 113 | 12.8 | 3 | 4 |
| 187 | 488646007 | 50S ribosomal protein L13 | 144 | 16.3 | 5 | 5 |
| 188 | 488646008 | 30S ribosomal protein S9 | 130 | 14.4 | 5 | 5 |
| 189 | 488646019 | phosphoenolpyruvate--protein phosphotransferase | 539 | 59.3 | 5 | 5 |
| 190 | 488646027 | butyrate kinase | 355 | 38.2 | 5 | 5 |
| 191 | 488646028 | ketol-acid reductoisomerase | 341 | 37.3 | 1 | 4 |
| 192 | 488646060 | glutamine--fructose-6-phosphate aminotransferase | 608 | 66.8 | 4 | 5 |
| 193 | 488646082 | galactose-6-phosphate isomerase | 172 | 19.2 | 4 | 1 |
| 194 | 488646101 | 2-C-methyl-D-erythritol 2,4-cyclodiphosphate synthase | 155 | 16.8 | 3 | 5 |
| 195 | 488646128 | redox-sensing transcriptional repressor Rex | 211 | 23.7 | 4 | 5 |
| 196 | 488646129 | crotonase | 261 | 28.2 | 5 | 5 |
| 197 | 488646137 | molecular chaperone GroES | 94 | 10.2 | 3 | 3 |
| 198 | 488646138 | molecular chaperone GroEL | 542 | 57.9 | 5 | 5 |
| 199 | 488646147 | glucose-6-phosphate isomerase | 449 | 49.8 | 5 | 5 |
| 200 | 488646186 | DNA polymerase III subunit beta | 368 | 41.5 | 5 | 5 |
| 201 | 488646194 | tRNA uridine-5-carboxymethylaminomethyl(34) synthesis enzyme MnmG | 628 | 69.8 | 2 | 4 |
| 202 | 488646195 | 16S rRNA methyltransferase G | 239 | 27.1 | 3 | 1 |
| 203 | 488646206 | 30S ribosomal protein S6 | 95 | 10.9 | 4 | 4 |
| 204 | 488646212 | ATP-dependent protease, Lon family | 631 | 69.9 | 3 | 2 |
| 205 | 488646220 | acyl-ACP thioesterase | 252 | 29.7 | 3 | 3 |
| 206 | 488646230 | UDP-N-acetylglucosamine 1-carboxyvinyltransferase | 421 | 45.3 | 4 | 5 |
| 207 | 488646232 | hypothetical protein | 167 | 19.4 | 5 | 5 |
| 208 | 488646239 | transferase | 243 | 27.3 | 5 | 5 |
| 209 | 488646243 | PTS sugar transporter subunit IIB | 101 | 10.7 | 4 | 4 |
| 210 | 488646257 | flagellar motor protein MotA | 260 | 28.1 | 3 | 4 |
| 211 | 488646320 | ferredoxin-NADP+ reductase subunit alpha | 295 | 32.4 | 5 | 4 |
| 212 | 488646337 | glycine--tRNA ligase | 463 | 53.4 | 4 | 5 |
| 213 | 488646339 | lysine--tRNA ligase | 501 | 57.5 | 5 | 5 |
| 214 | 488646340 | transcription elongation factor GreA | 161 | 18.0 | 5 | 5 |
| 215 | 488646343 | formate--tetrahydrofolate ligase | 556 | 59.9 | 4 | 5 |
| 216 | 488646345 | hypoxanthine phosphoribosyltransferase | 179 | 20.1 | 4 | 5 |
| 217 | 488646349 | RNA-binding protein S1 | 134 | 15.0 | 2 | 1 |
| 218 | 488646354 | transcriptional regulator | 91 | 10.2 | 4 | 4 |
| 219 | 488646355 | nucleoside triphosphate pyrophosphohydrolase | 483 | 55.1 | 5 | 5 |
| 220 | 488646364 | bifunctional N-acetylglucosamine-1-phosphate uridyltransferase/glucosamine-1-phosphate acetyltransferase | 456 | 50.0 | 4 | 5 |
| 221 | 488646366 | pur operon repressor | 271 | 29.8 | 1 | 2 |
| 222 | 488646367 | UDP-N-acetylmuramate--L-alanine ligase | 461 | 50.8 | 4 | 4 |
| 223 | 488646377 | anaerobic ribonucleoside triphosphate reductase | 702 | 79.6 | 3 | 4 |
| 224 | 488646378 | hypothetical protein | 281 | 30.7 | 2 | 5 |
| 225 | 488646382 | sodium:proton antiporter | 281 | 30.7 | 5 | 4 |
| 226 | 488646387 | 16S rRNA (adenine(1518)-N(6)/adenine(1519)-N(6))-dimethyltransferase | 281 | 31.9 | 4 | 4 |
| 227 | 488646395 | Xaa-Pro aminopeptidase | 415 | 47.5 | 3 | 4 |
| 228 | 488646401 | LacI family transcriptional regulator | 332 | 36.8 | 5 | 5 |
| 229 | 488646439 | ferredoxin | 574 | 63.0 | 4 | 5 |
| 230 | 488646450 | serine--tRNA ligase | 426 | 48.6 | 5 | 5 |
| 231 | 488646501 | PTS glucose transporter subunit IIA | 187 | 20.0 | 4 | 5 |
| 232 | 488646512 | 50S ribosomal protein L11 | 141 | 14.9 | 5 | 5 |
| 233 | 488646513 | 50S ribosomal protein L1 | 229 | 24.5 | 5 | 5 |
| 234 | 488646514 | 50S ribosomal protein L10 | 163 | 17.9 | 5 | 5 |
| 235 | 488646519 | 30S ribosomal protein S7 | 156 | 17.6 | 5 | 5 |
| 236 | 489464446 | hypothetical protein | 144 | 16.8 | 4 | 5 |
| 237 | 489501215 | flavin reductase | 165 | 19.5 | 4 | 5 |
| 238 | 489501274 | glucose-1-phosphate adenylyltransferase | 387 | 43.1 | 5 | 5 |
| 239 | 489501277 | glucose-1-phosphate adenylyltransferase subunit GlgD | 368 | 41.6 | 3 | 5 |
| 240 | 489501280 | starch synthase | 479 | 55.4 | 3 | 3 |
| 241 | 489501346 | aspartate-semialdehyde dehydrogenase | 361 | 40.2 | 5 | 5 |
| 242 | 489501444 | pyruvate carboxylase | 1148 | 128.0 | 4 | 5 |
| 243 | 489501501 | hypothetical protein | 1388 | 160.6 | 4 | 5 |
| 244 | 489501621 | hypothetical protein | 842 | 92.4 | 4 | 4 |
| 245 | 489501622 | hypothetical protein | 157 | 18.2 | 2 | 3 |
| 246 | 489501674 | DNA topoisomerase I | 697 | 79.3 | 3 | 4 |
| 247 | 489501736 | 1-phosphofructokinase | 301 | 33.0 | 5 | 5 |
| 248 | 489501744 | PTS fructose transporter subunit IIC | 640 | 67.4 | 3 | 3 |
| 249 | 489501773 | malate dehydrogenase | 391 | 42.1 | 4 | 4 |
| 250 | 489501774 | chromosomal replication initiation protein DnaA | 450 | 50.9 | 3 | 3 |
| 251 | 489501787 | DNA gyrase subunit B | 637 | 71.3 | 4 | 5 |
| 252 | 489501807 | NADH oxidase | 443 | 49.1 | 5 | 5 |
| 253 | 489501817 | hypothetical protein | 140 | 15.7 | 2 | 3 |
| 254 | 489501819 | valine--tRNA ligase | 880 | 100.7 | 5 | 5 |
| 255 | 489501869 | phenylalanine--tRNA ligase subunit alpha | 339 | 38.1 | 3 | 4 |
| 256 | 489501903 | sugar-binding protein | 425 | 45.6 | 5 | 3 |
| 257 | 489501910 | glycerol dehydrogenase | 363 | 38.7 | 4 | 5 |
| 258 | 489501915 | potassium transporter Trk | 221 | 24.2 | 2 | 1 |
| 259 | 489501922 | 7-cyano-7-deazaguanine synthase QueC | 218 | 24.5 | 2 | 3 |
| 260 | 489501962 | phenylalanine--tRNA ligase subunit beta | 792 | 88.2 | 5 | 5 |
| 261 | 489502024 | aspartate aminotransferase family protein | 429 | 47.4 | 4 | 5 |
| 262 | 489502034 | hypothetical protein | 174 | 20.3 | 4 | 4 |
| 263 | 489502046 | delta-aminolevulinic acid dehydratase | 322 | 36.0 | 4 | 4 |
| 264 | 489502147 | dTDP-4-dehydrorhamnose 3,5-epimerase | 183 | 20.9 | 5 | 5 |
| 265 | 489502151 | dTDP-glucose 4,6-dehydratase | 339 | 38.8 | 4 | 2 |
| 266 | 489502155 | metallophosphoesterase | 258 | 30.1 | 2 | 3 |
| 267 | 489502158 | glucose-1-phosphate thymidylyltransferase | 293 | 32.6 | 5 | 5 |
| 268 | 489502173 | aminotransferase DegT | 378 | 43.2 | 5 | 5 |
| 269 | 489502184 | glycosyl hydrolase family 32 | 339 | 36.2 | 3 | 1 |
| 270 | 489502201 | hypothetical protein | 224 | 24.9 | 3 | 4 |
| 271 | 489502203 | UDP-glucose 4-epimerase | 283 | 32.1 | 5 | 4 |
| 272 | 489502278 | hypothetical protein | 179 | 20.7 | 3 | 1 |
| 273 | 489502299 | hypothetical protein | 99 | 11.7 | 2 | 2 |
| 274 | 489502310 | pyridine nucleotide-disulfide oxidoreductase | 416 | 45.8 | 5 | 5 |
| 275 | 489502365 | glycerol kinase | 499 | 55.5 | 5 | 5 |
| 276 | 489502374 | tail fiber assembly protein | 159 | 17.9 | 1 | 3 |
| 277 | 489502503 | dihydroorotate dehydrogenase electron transfer subunit | 247 | 27.4 | 2 | 3 |
| 278 | 489502510 | GTP-binding protein YchF | 365 | 40.8 | 5 | 4 |
| 279 | 489502520 | phosphoglucomutase | 575 | 64.5 | 5 | 5 |
| 280 | 489502531 | hypothetical protein | 681 | 79.3 | 3 | 5 |
| 281 | 489502553 | formiminotransferase-cyclodeaminase | 214 | 24.2 | 3 | 4 |
| 282 | 489502574 | 50S ribosomal protein L25 | 190 | 20.8 | 5 | 5 |
| 283 | 489502629 | FprA family A-type flavoprotein | 388 | 43.6 | 5 | 5 |
| 284 | 489502642 | alpha,alpha-phosphotrehalase | 552 | 64.7 | 5 | 5 |
| 285 | 489502748 | phosphopyruvate hydratase | 430 | 47.0 | 2 | 4 |
| 286 | 489502792 | acetate kinase | 398 | 43.4 | 5 | 5 |
| 287 | 489502806 | hypothetical protein | 132 | 15.6 | 5 | 4 |
| 288 | 489502812 | V-type ATP synthase subunit F | 103 | 11.3 | 3 | 4 |
| 289 | 489502814 | V-type ATP synthase subunit D | 213 | 24.6 | 3 | 1 |
| 290 | 489502824 | HPr kinase/phosphorylase | 303 | 34.4 | 2 | 3 |
| 291 | 489502826 | ATP synthase subunit E | 196 | 21.8 | 5 | 4 |
| 292 | 489502828 | aminopeptidase | 464 | 51.6 | 4 | 4 |
| 293 | 489502911 | elongation factor G | 688 | 75.6 | 5 | 5 |
| 294 | 489502929 | cell wall hydrolase | 670 | 72.8 | 3 | 1 |
| 295 | 489502964 | RNA helicase | 584 | 68.0 | 3 | 2 |
| 296 | 489502966 | ABC transporter ATP-binding protein | 238 | 26.2 | 3 | 4 |
| 297 | 489502970 | peptidase T | 408 | 45.0 | 4 | 5 |
| 298 | 489502974 | transporter | 468 | 51.7 | 2 | 1 |
| 299 | 489502979 | transaldolase | 226 | 25.1 | 3 | 3 |
| 300 | 489502997 | 4-hydroxy-3-methylbut-2-en-1-yl diphosphate synthase | 349 | 37.9 | 2 | 5 |
| 301 | 489503004 | elongation factor Ts | 305 | 33.6 | 5 | 5 |
| 302 | 489503006 | transcription termination/antitermination protein NusA | 384 | 43.0 | 5 | 5 |
| 303 | 489503013 | polyribonucleotide nucleotidyltransferase | 701 | 77.0 | 5 | 5 |
| 304 | 489503028 | nicotinate phosphoribosyltransferase | 492 | 56.1 | 4 | 5 |
| 305 | 489503040 | helicase-exonuclease AddAB subunit AddA | 1252 | 144.2 | 1 | 2 |
| 306 | 489503149 | glycosyl transferase | 201 | 23.1 | 2 | 2 |
| 307 | 489503158 | UDP-N-acetyl glucosamine 2-epimerase | 354 | 39.2 | 3 | 5 |
| 308 | 489503213 | oxidoreductase | 271 | 31.2 | 5 | 5 |
| 309 | 489503218 | PTS sugar transporter subunit IIB | 102 | 11.0 | 3 | 2 |
| 310 | 489503221 | glycosyltransferase WbuB | 406 | 46.0 | 1 | 2 |
| 311 | 489503272 | UDP-N-acetylmuramoyl-L-alanyl-D-glutamate--2,6-diaminopimelate ligase | 486 | 54.5 | 4 | 4 |
| 312 | 489503275 | cell division protein DivIVA | 203 | 23.9 | 4 | 3 |
| 313 | 489503325 | bifunctional 5,10-methylene-tetrahydrofolate dehydrogenase/5,10-methylene-tetrahydrofolate cyclohydrolase | 282 | 30.7 | 2 | 4 |
| 314 | 489503330 | UDP-N-acetylmuramoyl-tripeptide--D-alanyl-D-alanine ligase | 454 | 50.6 | 2 | 3 |
| 315 | 489503443 | chemotaxis protein | 570 | 63.3 | 5 | 5 |
| 316 | 489503455 | nitroreductase | 173 | 19.8 | 2 | 5 |
| 317 | 489503456 | glucokinase | 313 | 33.2 | 5 | 5 |
| 318 | 489503464 | saccharopine dehydrogenase | 399 | 44.8 | 3 | 3 |
| 319 | 489503534 | 6-phosphofructokinase | 318 | 33.8 | 5 | 5 |
| 320 | 489503582 | ATPase AAA | 703 | 78.9 | 5 | 4 |
| 321 | 489503598 | hydrolase | 225 | 24.2 | 1 | 2 |
| 322 | 489503637 | bifunctional glutamate--cysteine ligase/glutathione synthetase | 775 | 89.6 | 4 | 3 |
| 323 | 489503664 | hypothetical protein | 190 | 22.6 | 1 | 3 |
| 324 | 489503695 | hypothetical protein | 337 | 36.5 | 2 | 5 |
| 325 | 489503725 | butanediol dehydrogenase | 358 | 38.5 | 3 | 3 |
| 326 | 489503786 | flagellar export chaperone FliS | 125 | 14.5 | 4 | 4 |
| 327 | 489503800 | elongation factor 4 | 601 | 67.1 | 3 | 4 |
| 328 | 489503802 | hypothetical protein | 143 | 16.7 | 4 | 5 |
| 329 | 489503822 | DNA topoisomerase IV subunit A | 971 | 110.3 | 3 | 4 |
| 330 | 489503830 | deoxyuridine 5'-triphosphate nucleotidohydrolase | 145 | 15.8 | 2 | 3 |
| 331 | 489503834 | hypothetical protein | 112 | 13.2 | 4 | 4 |
| 332 | 489503849 | hypothetical protein | 241 | 27.6 | 2 | 2 |
| 333 | 489503852 | phosphatase | 239 | 26.4 | 1 | 4 |
| 334 | 489503854 | chemotaxis protein CheW | 149 | 17.0 | 4 | 5 |
| 335 | 489503874 | chemotaxis protein CheR | 255 | 29.9 | 2 | 4 |
| 336 | 489503883 | flagellar hook-associated protein FlgK | 610 | 66.5 | 5 | 4 |
| 337 | 489503886 | flagellin | 270 | 29.0 | 5 | 5 |
| 338 | 489503907 | flagellar assembly protein FliW | 141 | 16.4 | 2 | 3 |
| 339 | 489503913 | flagellar motor switch protein FliM | 332 | 37.4 | 3 | 1 |
| 340 | 489503948 | ATP-dependent chaperone ClpB | 871 | 98.3 | 5 | 5 |
| 341 | 489503956 | inorganic pyrophosphatase | 548 | 60.3 | 5 | 5 |
| 342 | 489504006 | D-alanine--poly(phosphoribitol) ligase | 505 | 57.2 | 5 | 5 |
| 343 | 489504018 | NAD-dependent dehydratase | 328 | 37.2 | 3 | 3 |
| 344 | 489504026 | chemotaxis protein CheA | 684 | 76.0 | 5 | 5 |
| 345 | 489504043 | 4-methyl-5(B-hydroxyethyl)-thiazole monophosphate biosynthesis protein | 184 | 20.1 | 3 | 3 |
| 346 | 489504047 | UBA/TS-N domain | 210 | 23.2 | 5 | 3 |
| 347 | 489504053 | oligoendopeptidase F | 595 | 68.9 | 4 | 5 |
| 348 | 489504055 | pyruvate, phosphate dikinase | 875 | 96.6 | 5 | 5 |
| 349 | 489504059 | ribosomal protein L11 methyltransferase | 314 | 34.5 | 2 | 3 |
| 350 | 489504061 | motility protein A | 272 | 29.1 | 3 | 4 |
| 351 | 489504077 | flavodoxin | 141 | 15.8 | 4 | 4 |
| 352 | 489504086 | flagellar motor switch phosphatase FliY | 412 | 45.0 | 5 | 5 |
| 353 | 489504115 | 3-methyl-2-oxobutanoate hydroxymethyltransferase | 275 | 29.8 | 1 | 5 |
| 354 | 489504117 | hydrogenase expression protein HypA | 480 | 52.7 | 5 | 2 |
| 355 | 489504152 | 4-hydroxy-tetrahydrodipicolinate reductase | 252 | 27.6 | 5 | 5 |
| 356 | 489504162 | peptide ABC transporter substrate-binding protein | 551 | 60.5 | 5 | 5 |
| 357 | 489504246 | hypothetical protein | 145 | 17.4 | 3 | 2 |
| 358 | 489504250 | hypothetical protein | 144 | 15.9 | 3 | 3 |
| 359 | 489504253 | hypothetical protein | 348 | 39.5 | 3 | 3 |
| 360 | 489504271 | DNA primase | 856 | 98.8 | 3 | 3 |
| 361 | 489504273 | hypothetical protein | 131 | 14.6 | 5 | 3 |
| 362 | 489504275 | tRNA preQ1(34) S-adenosylmethionine ribosyltransferase-isomerase QueA | 341 | 39.0 | 3 | 3 |
| 363 | 489504278 | hypothetical protein | 389 | 43.0 | 3 | 3 |
| 364 | 489504281 | phage portal protein | 201 | 23.1 | 3 | 3 |
| 365 | 489504293 | hypothetical protein | 142 | 16.7 | 5 | 3 |
| 366 | 489504296 | hypothetical protein | 356 | 39.2 | 3 | 3 |
| 367 | 489504297 | hypothetical protein | 1111 | 120.7 | 3 | 3 |
| 368 | 489504314 | hypothetical protein | 705 | 80.4 | 3 | 2 |
| 369 | 489504443 | GTPase HflX | 596 | 66.9 | 3 | 3 |
| 370 | 489504484 | penicillin-binding protein 1A | 837 | 91.9 | 2 | 2 |
| 371 | 489504545 | endothelin-converting protein | 676 | 77.3 | 5 | 5 |
| 372 | 489504564 | 3-phosphoglycerate dehydrogenase | 302 | 32.3 | 5 | 4 |
| 373 | 489504570 | 5'-methylthioadenosine/S-adenosylhomocysteine nucleosidase | 230 | 25.1 | 5 | 5 |
| 374 | 489504589 | transcription antiterminator BglG | 284 | 32.5 | 2 | 2 |
| 375 | 489504590 | nucleoid-associated protein, YbaB/EbfC family | 113 | 12.0 | 4 | 4 |
| 376 | 489504639 | hypothetical protein | 418 | 48.0 | 3 | 2 |
| 377 | 489504644 | fructokinase | 314 | 34.0 | 5 | 4 |
| 378 | 489504650 | bifunctional acetaldehyde-CoA/alcohol dehydrogenase | 876 | 95.6 | 5 | 5 |
| 379 | 489504713 | peptidase M20 | 470 | 51.2 | 3 | 5 |
| 380 | 489504716 | cystathionine gamma-synthase | 387 | 42.6 | 3 | 2 |
| 381 | 489504751 | hypothetical protein | 215 | 23.7 | 3 | 2 |
| 382 | 489504798 | preprotein translocase subunit SecY | 428 | 46.7 | 3 | 3 |
| 383 | 489504839 | S-ribosylhomocysteine lyase | 159 | 18.0 | 4 | 5 |
| 384 | 489504864 | 50S ribosomal protein L7/L12 | 121 | 12.5 | 4 | 4 |
| 385 | 489504865 | DNA-directed RNA polymerase subunit beta' | 1178 | 131.6 | 5 | 5 |
| 386 | 489504866 | DNA-directed RNA polymerase subunit beta | 1235 | 138.5 | 5 | 5 |
| 387 | 489504938 | transcriptional repressor | 154 | 18.0 | 5 | 5 |
| 388 | 489504941 | hypothetical protein | 416 | 47.7 | 3 | 5 |
| 389 | 489504960 | toxin | 748 | 84.6 | 5 | 5 |
| 390 | 489504961 | peptidase M27 | 1163 | 136.7 | 5 | 5 |
| 391 | 489504979 | GTP-binding protein TypA | 608 | 67.7 | 3 | 2 |
| 392 | 489504981 | acetyl-CoA carboxylase biotin carboxylase subunit | 450 | 50.3 | 4 | 5 |
| 393 | 489505030 | alanine--tRNA ligase | 879 | 98.1 | 5 | 5 |
| 394 | 489505037 | acetyl-CoA carboxylase, biotin carboxyl carrier protein | 168 | 18.7 | 4 | 4 |
| 395 | 489505052 | iron-sulfur cluster assembly scaffold protein | 144 | 16.0 | 4 | 3 |
| 396 | 489505096 | ribonuclease J | 555 | 61.7 | 4 | 5 |
| 397 | 489505139 | glutamate synthase subunit alpha | 1526 | 170.1 | 4 | 5 |
| 398 | 489505309 | iron-only hydrogenase system regulator | 81 | 9.0 | 2 | 3 |
| 399 | 489505313 | galactokinase | 389 | 43.0 | 5 | 5 |
| 400 | 489505316 | nucleoside kinase | 551 | 63.9 | 2 | 4 |
| 401 | 489505346 | leucine--tRNA ligase | 816 | 92.9 | 5 | 5 |
| 402 | 489505355 | N-acetylmannosamine-6-phosphate 2-epimerase | 232 | 25.4 | 5 | 2 |
| 403 | 489505363 | tyrosine--tRNA ligase | 406 | 45.8 | 5 | 5 |
| 404 | 489505375 | L-lactate dehydrogenase | 312 | 33.8 | 5 | 5 |
| 405 | 489505394 | mannose-6-phosphate isomerase | 326 | 37.0 | 3 | 2 |
| 406 | 489505398 | UTP--glucose-1-phosphate uridylyltransferase | 295 | 33.2 | 4 | 5 |
| 407 | 489505407 | galactose-1-phosphate uridylyltransferase | 497 | 57.5 | 4 | 4 |
| 408 | 489505442 | 3-dehydroquinase | 254 | 28.1 | 2 | 4 |
| 409 | 489505521 | 2-Cys peroxiredoxin | 163 | 18.1 | 3 | 1 |
| 410 | 489505527 | tryptophan--tRNA ligase | 338 | 38.2 | 3 | 5 |
| 411 | 489505568 | sulfite reductase | 224 | 24.1 | 3 | 3 |
| 412 | 489505580 | protease | 102 | 11.4 | 2 | 4 |
| 413 | 489505585 | HD family phosphohydrolase | 349 | 40.0 | 3 | 2 |
| 414 | 489505662 | maltodextrin glucosidase | 447 | 52.6 | 3 | 3 |
| 415 | 489505693 | hypothetical protein | 252 | 29.6 | 5 | 5 |
| 416 | 489505705 | BMP family ABC transporter substrate-binding protein | 370 | 39.3 | 5 | 5 |
| 417 | 489505722 | molecular chaperone HtpG | 644 | 75.2 | 5 | 5 |
| 418 | 489505750 | hypothetical protein | 463 | 52.6 | 3 | 4 |
| 419 | 489505787 | thioether cross-link-forming SCIFF peptide maturase | 453 | 52.5 | 2 | 3 |
| 420 | 489505820 | 2-hydroxy-acid oxidase | 469 | 51.8 | 5 | 5 |
| 421 | 489505821 | GMP synthetase | 513 | 56.6 | 5 | 5 |
| 422 | 489505827 | 4-alpha-glucanotransferase | 520 | 60.5 | 4 | 5 |
| 423 | 489505831 | DNA ligase (NAD(+)) LigA | 663 | 74.6 | 5 | 4 |
| 424 | 489505835 | maltodextrin phosphorylase | 752 | 86.5 | 5 | 5 |
| 425 | 489505841 | ribonucleotide-diphosphate reductase subunit beta | 344 | 40.6 | 4 | 4 |
| 426 | 489505845 | PTS glucitol transporter subunit IIA | 469 | 51.4 | 3 | 2 |
| 427 | 489505855 | ribonucleotide-diphosphate reductase subunit alpha | 743 | 85.4 | 4 | 4 |
| 428 | 489505871 | acyl-CoA dehydrogenase | 379 | 41.0 | 5 | 5 |
| 429 | 489505884 | phosphate butyryltransferase | 302 | 32.5 | 5 | 5 |
| 430 | 489505902 | aspartyl/glutamyl-tRNA amidotransferase subunit A | 485 | 52.7 | 5 | 5 |
| 431 | 489505947 | fructose-bisphosphate aldolase | 284 | 31.7 | 2 | 3 |
| 432 | 489505963 | PTS fructose transporter subunit IIBC | 155 | 17.7 | 3 | 1 |
| 433 | 489505967 | threonine--tRNA ligase | 643 | 73.8 | 5 | 5 |
| 434 | 489505972 | acyltransferase | 872 | 100.8 | 5 | 5 |
| 435 | 489505976 | aspartyl/glutamyl-tRNA amidotransferase subunit B | 476 | 53.6 | 5 | 5 |
| 436 | 489505984 | IMP dehydrogenase | 484 | 52.1 | 4 | 5 |
| 437 | 489506024 | alpha-glycosidase | 576 | 68.3 | 4 | 3 |
| 438 | 489506025 | asparaginyl/glutamyl-tRNA amidotransferase subunit C | 96 | 10.9 | 4 | 4 |
| 439 | 489506027 | ABC transporter substrate-binding protein | 438 | 47.7 | 5 | 5 |
| 440 | 489506059 | signal peptide peptidase SppA | 331 | 36.0 | 3 | 4 |
| 441 | 489506062 | N-6 DNA methylase | 642 | 73.5 | 2 | 5 |
| 442 | 489506066 | multidrug transporter | 428 | 48.3 | 3 | 3 |
| 443 | 489506071 | hypothetical protein | 186 | 21.7 | 2 | 4 |
| 444 | 489506077 | PTS fructose transporter subunit IIB | 101 | 11.4 | 4 | 3 |
| 445 | 489506081 | 8-oxoguanine DNA glycosylase | 309 | 36.4 | 5 | 5 |
| 446 | 489506082 | phosphoglucosamine mutase | 448 | 48.5 | 2 | 5 |
| 447 | 489506104 | electron transfer flavoprotein subunit alpha | 335 | 35.8 | 5 | 5 |
| 448 | 489506129 | electron transfer flavoprotein subunit beta | 259 | 27.7 | 5 | 5 |
| 449 | 489506204 | peptidylprolyl isomerase | 247 | 27.8 | 5 | 5 |
| 450 | 489506424 | NAD(+) synthase | 632 | 71.5 | 3 | 3 |
| 451 | 489506425 | branched chain amino acid aminotransferase | 342 | 38.0 | 1 | 2 |
| 452 | 489506509 | PTS mannose/fructose/sorbose transporter subunit IIC | 268 | 27.2 | 5 | 5 |
| 453 | 489506558 | alveolysin | 513 | 57.3 | 3 | 2 |
| 454 | 489506590 | thioredoxin reductase | 287 | 31.9 | 5 | 3 |
| 455 | 489506753 | NAD(P)-dependent oxidoreductase | 326 | 37.5 | 3 | 1 |
| 456 | 489506790 | triose-phosphate isomerase | 248 | 26.5 | 5 | 5 |
| 457 | 489506809 | 30S ribosomal protein S1 | 383 | 42.9 | 5 | 4 |
| 458 | 489506819 | hypothetical protein | 233 | 26.1 | 4 | 4 |
| 459 | 489506854 | tRNA (uridine(34)/cytosine(34)/5 carboxymethylaminomethyluridine(34)-2'-O) methyltransferase TrmL | 154 | 17.7 | 5 | 5 |
| 460 | 489506862 | phosphoglycerate kinase | 391 | 41.7 | 5 | 5 |
| 461 | 489506869 | endoribonuclease L-PSP | 124 | 13.5 | 2 | 4 |
| 462 | 489506885 | cysteine synthase A | 303 | 32.2 | 5 | 5 |
| 463 | 489506887 | RDD family protein, partial | 136 | 15.7 | 2 | 3 |
| 464 | 489506930 | 23S rRNA (uracil-5-)-methyltransferase RumA | 451 | 50.8 | 4 | 4 |
| 465 | 489506932 | preprotein translocase subunit SecA | 902 | 101.2 | 5 | 5 |
| 466 | 489506945 | 1-acyl-sn-glycerol-3-phosphate acyltransferase | 234 | 26.2 | 2 | 3 |
| 467 | 489506971 | RNA-binding transcriptional accessory protein | 723 | 80.7 | 2 | 4 |
| 468 | 489507017 | hypothetical protein | 1215 | 138.8 | 3 | 4 |
| 469 | 489507078 | hypothetical protein | 1202 | 140.2 | 3 | 5 |
| 470 | 489507158 | DNA-binding protein | 261 | 28.8 | 2 | 4 |
| 471 | 489507226 | ATP--guanido phosphotransferase | 337 | 38.9 | 2 | 4 |
| 472 | 489507233 | ser/threonine protein phosphatase | 232 | 26.0 | 3 | 5 |
| 473 | 489507254 | hypothetical protein | 123 | 14.2 | 3 | 3 |
| 474 | 489507261 | UDP-N-acetylmuramoylalanine--D-glutamate ligase | 458 | 51.3 | 5 | 4 |
| 475 | 489507270 | two-component sensor histidine kinase | 294 | 34.1 | 3 | 2 |
| 476 | 489507271 | proline--tRNA ligase | 570 | 63.6 | 5 | 5 |
| 477 | 489507272 | carbamoyl phosphate synthase large subunit | 1070 | 119.1 | 3 | 3 |
| 478 | 489507278 | aconitate hydratase | 642 | 69.1 | 3 | 5 |
| 479 | 489507280 | hypothetical protein | 276 | 31.9 | 2 | 4 |
| 480 | 489507281 | galactose mutarotase | 291 | 33.8 | 2 | 3 |
| 481 | 489507283 | methionine--tRNA ligase | 647 | 73.3 | 5 | 5 |
| 482 | 489507299 | asparagine--tRNA ligase | 464 | 53.2 | 5 | 5 |
| 483 | 489507313 | heme ABC transporter ATP-binding protein | 529 | 59.1 | 5 | 4 |
| 484 | 489507326 | transcription-repair coupling factor | 1166 | 134.1 | 4 | 5 |
| 485 | 489507366 | septation protein SpoVG | 91 | 10.2 | 4 | 4 |
| 486 | 489507387 | ATP-dependent Clp protease ATP-binding subunit ClpC | 814 | 92.3 | 4 | 5 |
| 487 | 489507409 | cysteine--tRNA ligase | 466 | 53.7 | 4 | 4 |
| 488 | 489507421 | glutamine synthetase | 632 | 71.8 | 5 | 5 |
| 489 | 489507440 | isoleucine--tRNA ligase | 1037 | 118.9 | 5 | 5 |
| 490 | 489507464 | thioredoxin-disulfide reductase | 311 | 34.4 | 3 | 2 |
| 491 | 489507502 | glucanase | 346 | 38.1 | 1 | 5 |
| 492 | 489507605 | hypothetical protein | 198 | 23.0 | 4 | 4 |
| 493 | 489507631 | hypothetical protein | 146 | 16.6 | 4 | 2 |
| 494 | 489507637 | tellurium resistance protein | 390 | 44.4 | 3 | 5 |
| 495 | 489507701 | methionine adenosyltransferase | 391 | 43.4 | 4 | 5 |
| 496 | 489507703 | F0F1 ATP synthase subunit delta | 179 | 21.4 | 5 | 5 |
| 497 | 489507712 | peptidoglycan-binding protein | 520 | 58.6 | 3 | 3 |
| 498 | 489507720 | peptidase U62 | 447 | 48.8 | 3 | 5 |
| 499 | 489507737 | ATP synthase subunit alpha | 504 | 55.1 | 5 | 5 |
| 500 | 489507751 | transcription termination factor Rho | 511 | 58.2 | 2 | 3 |
| 501 | 489507776 | PTS cellobiose transporter subunit IIA | 105 | 12.0 | 4 | 1 |
| 502 | 489508050 | methionyl-tRNA formyltransferase | 308 | 34.7 | 3 | 2 |
| 503 | 489508055 | 16S rRNA (cytosine(967)-C(5))-methyltransferase | 438 | 49.7 | 5 | 5 |
| 504 | 489508059 | protein phosphatase | 239 | 26.2 | 3 | 3 |
| 505 | 489508255 | type I-B CRISPR-associated protein Cas8b/Csh1 | 586 | 67.9 | 3 | 4 |
| 506 | 489508264 | nitrogenase cofactor biosynthesis protein NifB | 888 | 99.8 | 1 | 2 |
| 507 | 489508421 | formate acetyltransferase | 750 | 84.7 | 5 | 5 |
| 508 | 489508446 | CRISPR-associated protein | 316 | 35.5 | 5 | 5 |
| 509 | 489508481 | glutamine ABC transporter ATP-binding protein | 249 | 28.3 | 3 | 2 |
| 510 | 489508642 | 6-phospho-alpha-glucosidase | 444 | 50.2 | 4 | 3 |
| 511 | 489508885 | PspC family transcriptional regulator | 626 | 69.5 | 5 | 5 |
| 512 | 489508891 | UDP-N-acetylenolpyruvoylglucosamine reductase | 304 | 33.3 | 4 | 2 |
| 513 | 489508899 | signal peptidase I | 188 | 21.5 | 3 | 2 |
| 514 | 489508907 | diaminopimelate decarboxylase | 431 | 48.0 | 2 | 1 |
| 515 | 489508919 | ATP-dependent DNA helicase | 725 | 84.5 | 4 | 4 |
| 516 | 489508954 | pleiotropic regulatory protein DegT | 392 | 43.5 | 5 | 5 |
| 517 | 489508961 | amino acid ABC transporter | 272 | 29.1 | 5 | 3 |
| 518 | 489508962 | excinuclease ABC subunit A | 940 | 104.3 | 3 | 4 |
| 519 | 489508969 | UDP-N-acetyl-D-glucosamine dehydrogenase | 438 | 48.9 | 5 | 5 |
| 520 | 489508970 | M18 family aminopeptidase | 428 | 47.2 | 5 | 5 |
| 521 | 489508975 | hypothetical protein | 196 | 23.2 | 2 | 5 |
| 522 | 489508992 | collagenolytic protease | 446 | 49.4 | 4 | 5 |
| 523 | 489509023 | ABC transporter permease | 296 | 33.1 | 2 | 2 |
| 524 | 489509065 | flagellar hook protein | 688 | 75.1 | 4 | 5 |
| 525 | 489509085 | hypothetical protein | 177 | 20.2 | 4 | 5 |
| 526 | 489509164 | ribonuclease R | 734 | 84.6 | 4 | 5 |
| 527 | 489509269 | 50S ribosomal protein L3 | 209 | 22.8 | 5 | 5 |
| 528 | 489509381 | CoA-transferase | 510 | 56.0 | 2 | 3 |
| 529 | 489509402 | tail protein | 192 | 20.9 | 3 | 2 |
| 530 | 489509476 | hypothetical protein | 656 | 74.8 | 2 | 1 |
| 531 | 489509514 | two-component system response regulator | 291 | 32.2 | 2 | 3 |
| 532 | 489509564 | hypothetical protein | 586 | 67.7 | 5 | 5 |
| 533 | 489509663 | N-acetyltransferase | 204 | 23.8 | 2 | 3 |
| 534 | 489509757 | DNA topoisomerase III | 727 | 82.4 | 1 | 2 |
| 535 | 489509823 | hypothetical protein | 551 | 60.6 | 5 | 5 |
| 536 | 489509848 | uridine phosphorylase | 261 | 28.3 | 4 | 5 |
| 537 | 489509905 | xanthine phosphoribosyltransferase | 190 | 21.2 | 3 | 4 |
| 538 | 489509921 | 16S rRNA processing protein RimM | 166 | 19.3 | 2 | 4 |
| 539 | 489510010 | hypothetical protein | 138 | 16.0 | 2 | 3 |
| 540 | 489510022 | endopeptidase La | 775 | 87.0 | 2 | 3 |
| 541 | 489510029 | sporulation transcription factor Spo0A | 277 | 31.1 | 5 | 5 |
| 542 | 489510173 | amino acid lyase | 344 | 38.3 | 3 | 2 |
| 543 | 489510197 | hypothetical protein | 428 | 46.9 | 5 | 4 |
| 544 | 489510211 | sulfate transporter subunit | 344 | 38.2 | 4 | 3 |
| 545 | 489510215 | 30S ribosomal protein S2 | 233 | 26.3 | 5 | 5 |
| 546 | 489510263 | DNA recombination/repair protein RecA | 359 | 38.5 | 4 | 5 |
| 547 | 489510276 | hypothetical protein | 99 | 11.4 | 4 | 4 |
| 548 | 489510278 | hypothetical protein | 324 | 37.4 | 2 | 3 |
| 549 | 489510322 | translation initiation factor IF-3 | 173 | 19.8 | 2 | 5 |
| 550 | 489510327 | hypothetical protein | 848 | 99.4 | 2 | 3 |
| 551 | 489510332 | ABC transporter | 353 | 39.5 | 3 | 1 |
| 552 | 489510397 | ATP-dependent Clp protease ATP-binding subunit ClpX | 429 | 47.6 | 3 | 4 |
| 553 | 489510420 | L-asparaginase | 327 | 35.6 | 2 | 3 |
| 554 | 489510437 | preprotein translocase subunit SecF | 289 | 31.6 | 3 | 4 |
| 555 | 489510441 | hypothetical protein | 98 | 11.6 | 3 | 2 |
| 556 | 489510464 | ribonuclease Y | 513 | 57.8 | 3 | 3 |
| 557 | 489510493 | isocitrate dehydrogenase | 402 | 45.4 | 3 | 5 |
| 558 | 489510530 | endonuclease IV | 277 | 31.4 | 5 | 1 |
| 559 | 489510573 | DNA mismatch repair protein MutT | 202 | 23.0 | 2 | 5 |
| 560 | 489510619 | capsular polysaccharide biosynthesis protein | 391 | 44.3 | 5 | 5 |
| 561 | 489510620 | tRNA (N6-isopentenyl adenosine(37)-C2)-methylthiotransferase MiaB | 456 | 52.2 | 3 | 4 |
| 562 | 489510647 | UMP kinase | 237 | 25.7 | 3 | 5 |
| 563 | 489510659 | phosphoribosylformylglycinamidine synthase | 1248 | 138.1 | 3 | 3 |
| 564 | 489510856 | rubrerythrin | 180 | 19.8 | 5 | 5 |
| 565 | 489510996 | adenylosuccinate synthase | 428 | 47.4 | 5 | 5 |
| 566 | 489511000 | DNA gyrase subunit A | 835 | 93.1 | 5 | 5 |
| 567 | 489522987 | hypothetical protein | 127 | 14.8 | 4 | 1 |
| 568 | 567939684 | dihydroxyacetone kinase subunit L | 208 | 22.5 | 4 | 5 |
| 569 | 653633796 | PTS N-acetylgalactosamine transporter subunit IIB | 158 | 17.9 | 1 | 3 |
| 570 | 653633804 | transcription termination/antitermination protein NusG | 173 | 19.6 | 5 | 5 |
| 571 | 653633918 | serine protein kinase | 640 | 73.3 | 2 | 3 |
| 572 | 737822179 | adenylyl-sulfate reductase | 568 | 63.4 | 4 | 3 |
| 573 | 737822225 | phosphopentomutase | 396 | 44.3 | 4 | 4 |
| 574 | 754906167 | hypothetical protein | 188 | 22.2 | 5 | 5 |
| 575 | 906848776 | Chain D, Crystal Structure Of (s)-3-hydroxybutylryl-coa Dehydrogenase Form The N-butanol Sysnthesizing Bacterium, | 282 | 30.4 | 5 | 5 |
| 576 | 488644394 | precorrin-4 C(11)-methyltransferase | 252 | 28.0 | 2 | 2 |
| 577 | 488644703 | hypothetical protein | 91 | 10.3 | 1 | 2 |
| 578 | 489501515 | phospho-2-dehydro-3-deoxyheptonate aldolase | 342 | 38.9 | 2 | 2 |
| 579 | 489504787 | energy-coupling factor transporter ATPase | 287 | 32.0 | 1 | 2 |
| ***Proteins identified only in AE*** | | | | | | |
| 1 | 489503203 | 6-phospho-beta-glucosidase | 470 | 53.6 | 5 | 0 |
| 2 | 489503965 | flagellar basal body rod protein FlgG | 379 | 39.3 | 3 | 0 |
| 3 | 488646122 | hypothetical protein | 362 | 41.3 | 3 | 0 |
| 4 | 489504289 | hypothetical protein | 585 | 63.0 | 3 | 0 |
| 5 | 489509462 | hypothetical protein | 298 | 33.5 | 3 | 0 |
| 6 | 237656561 | hypothetical protein CLP_1654 | 256 | 27.3 | 3 | 0 |
| 7 | 489501666 | sulfate adenylyltransferase small subunit | 299 | 34.7 | 3 | 0 |
| 8 | 489503814 | UDP-N-acetylglucosamine 4,6-dehydratase (inverting) | 333 | 38.0 | 3 | 0 |
| ***Proteins identified only in AN*** | | | | | | |
| 1 | 488642862 | 5-(carboxyamino)imidazole ribonucleotide mutase | 159 | 17.2 | 0 | 4 |
| 2 | 489505689 | hypothetical protein | 342 | 38.8 | 0 | 4 |
| 3 | 489503008 | ribosome-recycling factor | 185 | 20.5 | 0 | 4 |
| 4 | 488642944 | 23S rRNA (adenine(2503)-C(2))-methyltransferase RlmN | 347 | 39.1 | 0 | 3 |
| 5 | 488645984 | 50S ribosomal protein L24 | 104 | 11.5 | 0 | 3 |
| 6 | 488643009 | aspartate kinase | 402 | 43.8 | 0 | 3 |
| 7 | 489501399 | hypothetical protein TPR repeat protein | 245 | 28.5 | 0 | 3 |
| 8 | 489507297 | hypothetical protein | 509 | 59.0 | 0 | 3 |
| 9 | 489504328 | PTS N-acetylgalactosamine transporter subunit IID | 281 | 30.8 | 0 | 3 |
| 10 | 489507369 | ribose-phosphate pyrophosphokinase | 321 | 34.9 | 0 | 3 |
| 11 | 489508929 | rRNA (cytidine-2'-O-)-methyltransferase | 281 | 32.0 | 0 | 3 |

**Accession:** NCBI entry; **Description:** name of the protein; **# AAs**: number of aminoacids; **MW**: molecular weight **AE:** number of spectral counts of the relative protein in AE condition; **AN**: number of spectral counts of the relative protein in AN condition.
